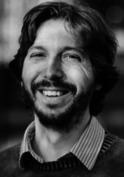# An Introduction to Model Compounds of Lignin Linking Motifs; Synthesis and Selection Considerations for Reactivity Studies

**DOI:** 10.1002/cssc.202000989

**Published:** 2020-07-09

**Authors:** Ciaran W. Lahive, Paul C. J. Kamer, Christopher S. Lancefield, Peter J. Deuss

**Affiliations:** ^1^ Department of Chemical Engineering (ENTEG) University of Groningen Nijenborgh 4 9747 AG Groningen Netherlands; ^2^ School of Chemistry and Biomedical Science Research Complex University of St. Andrews and EaStCHEM North Haugh St. Andrews Fife KY16 9ST United Kingdom; ^3^ Leibniz-Institut für Katalyse e.V. Albert-Einstein-Straße 29a 18059 Rostock Germany

**Keywords:** biomass, lignin, model compounds, organic synthesis

## Abstract

The development of fundamentally new valorization strategies for lignin plays a vital role in unlocking the true potential of lignocellulosic biomass as sustainable and economically compatible renewable carbon feedstock. In particular, new catalytic modification and depolymerization strategies are required. Progress in this field, past and future, relies for a large part on the application of synthetic model compounds that reduce the complexity of working with the lignin biopolymer. This aids the development of catalytic methodologies and in‐depth mechanistic studies and guides structural characterization studies in the lignin field. However, due to the volume of literature and the piecemeal publication of methodology, the choice of suitable lignin model compounds is far from straight forward, especially for those outside the field and lacking a background in organic synthesis. For example, in catalytic depolymerization studies, a balance between synthetic effort and fidelity compared to the actual lignin of interest needs to be found. In this Review, we provide a broad overview of the model compounds available to study the chemistry of the main native linking motifs typically found in lignins from woody biomass, the synthetic routes and effort required to access them, and discuss to what extent these represent actual lignin structures. This overview can aid researchers in their selection of the most suitable lignin model systems for the development of emerging lignin modification and depolymerization technologies, maximizing their chances of successfully developing novel lignin valorization strategies.

## Graphical abstract

**Model me right**: A review of lignin model compounds for the most abundant linking motifs is provided as well as the state‐of‐the‐art approaches for their synthesis. A framework is also offered for researches working in the field of lignin valorization on what considerations to take into account when choosing the right model for the right stage of methodological development to get the most out of every model compound study.

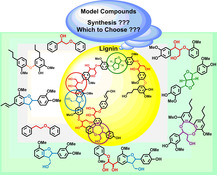

## 1 Introduction

### 1.1 Introduction to Lignin

To make our chemical industry sustainable, renewable carbon resources that can be applied as substitutes for finite fossil ones are required. The most abundant renewable source of carbon globally, apart from CO_2_, is lignocellulosic biomass, which includes wood and agricultural residues. These materials have therefore been identified as potential renewable substitutes for fossil resources.^1^, ^2^ There have been many new developments for the conversion of lignocellulosic materials towards chemical products and fuels. However, most of these, such as second‐generation bioethanol or furanics, extract value solely from the carbohydrate component, with the lignin component being treated as an undesired residue. Similarly, the more established paper industry focuses on high‐quality cellulose, which inherently leads to the generation of a large volume of low‐value lignin as a by‐product. Apart for some niche applications of lignosulfonate, these lignin residues are burned as a low‐value fuel, which is used to generate process heat. However, from a sustainability and an economic perspective more efficient resource utilization would be desirable.^3^ Therefore, value‐extraction from the lignin fraction of lignocellulosic biomass has become a major focus area. This includes the development of new fractionation methods as well as many elegant new catalytic methodologies for the depolymerization or modification of lignin to generate emerging lignin‐derived chemical products.^4^, ^5^ Such efforts are essential for providing additional revenue streams for bio‐refineries to boost their overall economic viability and competitiveness. To generate value from the lignin biopolymer, its highly complex chemical structure needs to be understood and dealt with.

**Figure 1:**
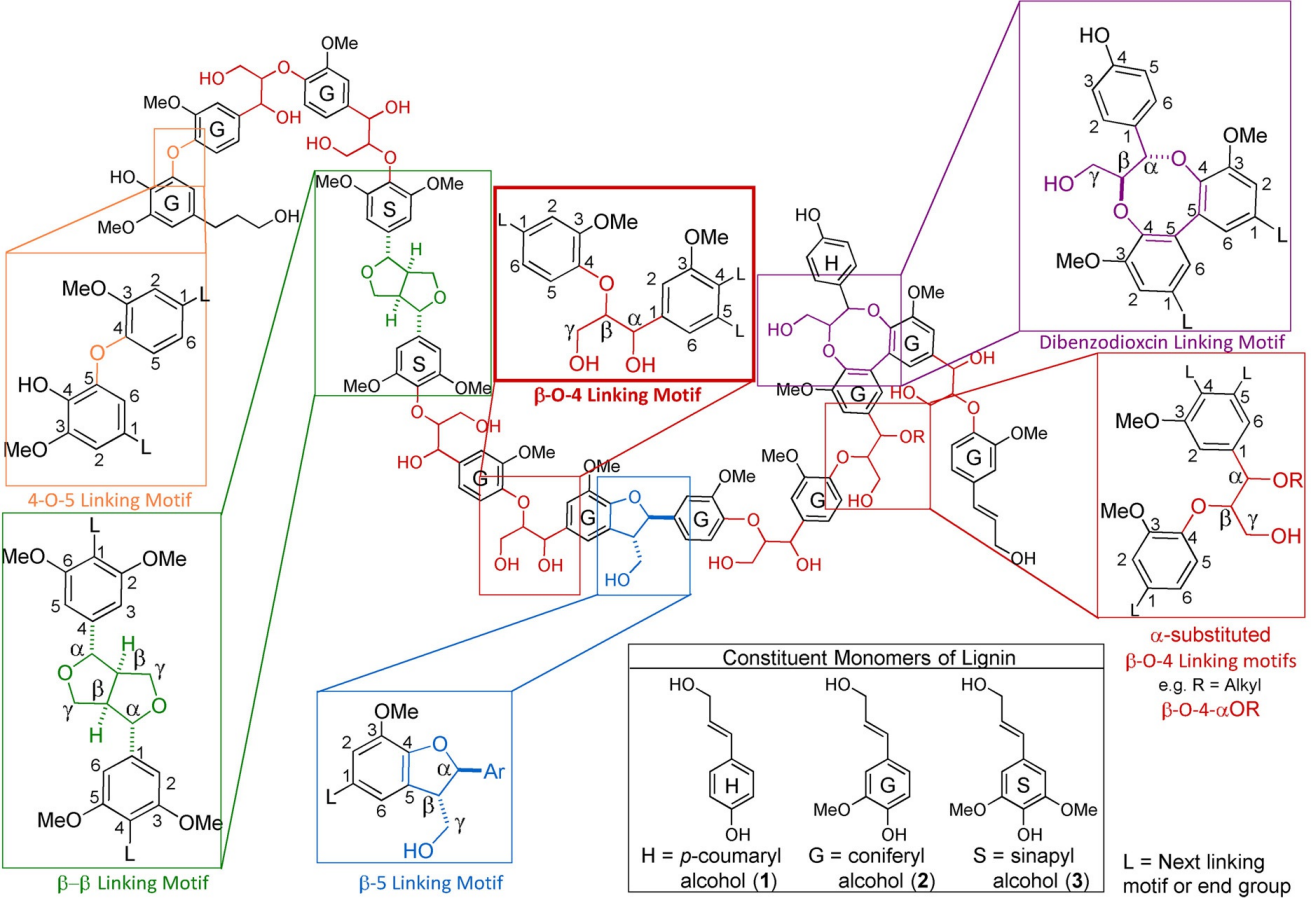
Illustrative lignin polymer structure representing typical lignins from woody biomass showing the most abundant aromatic units highlighted along with the most important linking motifs.

Approximately 450 million years ago, the first plants began to deposit lignin in their cell walls. This lignin evolved to play a key role in the defense of plants against pathogens and herbivores while also facilitating nutrient transportation and acting as a supportive structure. This allowed for an increase in the size of plants and contributed to their dominance of the terrestrial environment.^6^ The evolution of lignin biosynthesis has resulted in the formation of a highly complex, amorphous aromatic polymer consisting of phenylpropanoid subunits linked by a broad variety of C−O and C−C bonds. These originate, for the most part, from the combinatorial radical coupling of the monolignols: *p*‐coumaryl alcohol (**1**), coniferyl alcohol (**2**), and sinapyl alcohol (**3**) (Figure 1 bottom right).^3^, ^7^, ^8^ These three main monolignols provide aromatic units with different numbers of methoxy substituents referred to as *p*‐hydroxyphenyl (H), guaiacyl (G), and syringyl (S), respectively. The coupling reactions lead to a complex network of which an illustrative chemical representation showing the major linking motifs discussed in this Review is provided in Figure 1.

In planta, lignin has a highly complex structure that varies significantly between plant species and depends on plant age and numerous environmental factors.^9^ When lignin is separated from the cellulosic and hemicellulosic fractions of plant biomass, the structure invariably becomes even more complex as all isolation procedures induce chemical modifications in the structure. This complexity itself poses significant analytical challenges that are further exacerbated by lignin's high molecular weight. The primary strategy to mitigate these difficulties is by the use of model systems to study the lignin structure and reactivity. These model systems have been extensively developed and used, ranging from monoaromatic compounds to diaromatic linking motif model compounds, oligomeric model systems, up to fully synthetic dehydrogenation polymer (DHP) lignins. Although the selection of an appropriate model compound can be very important for the success or failure of a study, and its translation to real lignin chemistry is often a difficult choice as the literature on the topic is scattered and no comprehensive comparison of synthetic methods to access the compounds exists. This Review is aimed at providing this much needed overview by covering the types of native lignin linking motif model systems that have been developed and providing a discussion on the different synthetic methodologies that can be used to access them.

Many studies make use of phenol, anisole, or guaiacol as model compounds representing just the oxygenated aromatic motif. These model compounds can be useful when considering, for example, catalyst development for hydrodeoxygenation studies, where removal of aromatic substituents is the limiting step.^10^, ^11^, ^12^, ^13^, ^14^, ^15^ This Review, however, focuses on the study of lignin linking motif models and so will not be addressing the use of monomeric models. Thus, this overview will start with dimeric model compounds that contain one linking motif and is sectioned according to the type of motif. Further on, larger model structures bearing multiple linking motifs are also discussed. Finally, some general guidelines and considerations are provided for the selection of the right model compound for the type of research being undertaken, balancing the synthetic effort required against the fidelity of the model compounds. This should ultimately facilitate research studies to have the maximum impact in the field of lignin research.

### 1.2 Model compound naming

To follow discussions on lignin linking motifs and respective model compounds, it is important to understand the associated nomenclature. For the basic phenylpropanoid units that form lignin, shown in Figure 1, the carbon atoms in the aromatic rings are numbered 1–6, starting at the carbon atom attached to the propyl chain. The propyl chain is then most commonly numbered using the Greek letters α, β, and γ, starting at the carbon atom next to the aromatic ring, or, alternatively, by continuing the numerical sequence 7, 8, and 9 (the former will be used throughout this Review). Extending this to linking motifs, in most cases, the nomenclature used describes the bond formed during the key radical–radical coupling step^16^ but not the subsequent bonds formed during trapping of the resulting quinone methides. Thus, the β‐O‐4′ motif can be understood to connect the β carbon atom of one propyl chain to an oxygen atom at the 4 position of another aromatic unit. The prime (′) here denotes that the atom is from the second coupling unit; however, this descriptor is often omitted (as it is for the remainder of this review). Similarly, the terms β‐5′ and β‐β′ describe the motifs generated via coupling between the β‐position on one unit and the 5‐ or β‐positions on another unit. As these descriptors do not include all bonds formed during the coupling process, they are inherently ambiguous; however, β‐O‐4′ is usually used to describe arylglycerol‐β‐aryl ethers, β‐5′ for phenylcoumarans, and β‐β′ for resinols (Figure 1). For composite linking motifs such as dibenzodioxocins and spirodienones that involve the connection of more than two phenylpropanoid units, the system outlined above becomes impractical, and so naming follows the type of ring structure that is formed. For example, the 8‐membered ring of dibenzodioxocins contains 5‐5, α‐O‐4, and β‐O‐4 bonds. Moving to model compounds these naming conventions are typically retained, providing direct insight into the linking motif being modelled. It is important to note that the most commonly used names for the linking motifs are described here; however, other names are sometimes used in literature.

### 1.3 General application of lignin model compounds

Lignin model compounds are used for many reasons, but the primary ones being the study of structure and reactivity of lignin on a level of detail that is difficult to attain using lignin itself given its complexity and high molecular weight. Whilst there are clear benefits to using low molecular weight model compounds, there are also limitations, as summarized below.

**The benefits of the use of model compounds are**:

‐: simplification of the complex mixtures of products obtained from depolymerization reactions for ease of analysis
‐: use of a variety of model compounds of varying complexity allows development of a detailed understanding of the reaction mechanisms for degradation or modification
‐: the fate of individual linking motifs can be studied in isolation or simple combinations
‐: structural features formed via the modification of lignin linking motifs can be used to confirm the formation of new motifs in lignin

**The limitations of lignin model compounds are**:

‐: lack of the full complexity and variations of the chemical structure in different lignins
‐: different impurities than those found in isolated lignin streams
‐: the solubility constraints of isolated lignin polymers are not fully replicated
‐: the 3D environment created by the lignin polymer is not well represented
‐: the complexity of product streams and possible separation technology required are not replicated

There are many examples of the use of model compounds to study lignin. Recently, the main focus for model compound use has become the development of novel catalytic conversion methodologies. Here, however, the possibility frequently arises that a degradation/modification system that works efficiently in a model system may fail to be effective when applied to lignin. An example of this is the elegant hydrogen neutral Ru–Xantphos‐catalyzed lignin C−O cleavage methodology for the depolymerization of model β‐O‐4 motifs developed by Nichols et al.^17^ This methodology performed excellently on the initially tested simple dimeric and even polymeric lignin β‐O‐4 model systems, which lacked γ‐carbinol groups, but upon application of the methodology to higher‐fidelity β‐O‐4 model systems bearing a γ‐carbinol group, as found in lignin, the method proved ineffective. It was shown that the catalyst was deactivated via chelation of the Ru center by the oxidized γ‐carbinol and the α‐alcohol groups, resulting in a catalytically inactive acyl‐enolate complex. The γ‐carbinol group was not represented in the selected model compounds for the initial study, highlighting the importance of the lignin‐model choice.^18^ This also demonstrates that the better the model system can reflect the actual chemical structure of lignin, the more chance of successful translation of the chemistry to real lignin. However, as is discussed later, this is balanced by the investments in time, effort, and expertise required to obtain the appropriate model compound.

Given the complexity of lignin, there is a wide range of different model compounds that have been utilized to study its chemistry. As in the above example, studies most frequently employ models of the β‐O‐4 linking motif as it is almost universally the most commonly occurring structural unit across native lignins in various different types of biomass (Table 1). For other linkages their abundance is significantly lower and more variable. Therefore, the β‐O‐4 linking motif is often selected for the development of new catalytic lignin depolymerization/modification methodologies.^19^ Although it is the most obvious choice, it is important to note that the high abundance of β‐O‐4 linking motifs does not typically hold true for technical lignins as β‐aryl‐ethers can be significantly degraded during the fractionation process. This leads to the formation of a much wider variety of different linking motifs that are often of the C−C type and hard to degrade selectively.^3^ Such an array of structures is typically hard to capture in model compounds and therefore, the use of appropriate model compounds becomes more problematic.^20^, ^21^ Model compounds that represent other native lignin linking motifs are often used to study the effect of chemical processing on the lignin structure as a whole or for structural elucidation purposes.^20^, ^22^, ^23^, ^24^ In the remainder of this Review, the types of model compounds and synthetic methodologies to access these are provided based on the most common native linking motifs provided in Figure 1 and Table 1. Additionally, further discussion on model compound selection is provided to conclude this Review.

**Table 1:** Abundancies of some of the primary lignin linking motifs in softwoods, hardwoods, and grasses along with the monolignol ranges. Values quoted for lignin linking motifs are for abundance per 100 C_9_ units. Data taken from review articles.^3^, ^25^

Lignin	Linking motif [%]			5‐5^[a]^	4‐O‐5	Monomer [%]		
	β‐O‐4	β‐5	β‐β			H	G	S
softwood	45–50	9–12	2–6	5–7	2	<5	≈95	≈0
hardwood	60–62	3–11	3–12	<1	2	0–8	25–50	45–75
grasses	74–84	5–11	1–7	nd	nd	5‐‐35	35–80	20–55

[a] In the form of dibenzodioxocin.

## 2 Dimeric Model Compounds Representing Lignin Linking Motifs

### 2.1 β‐O‐4 type model compounds

#### 2.1.1 Standard β‐O‐4 model compounds.

**Figure 2:**
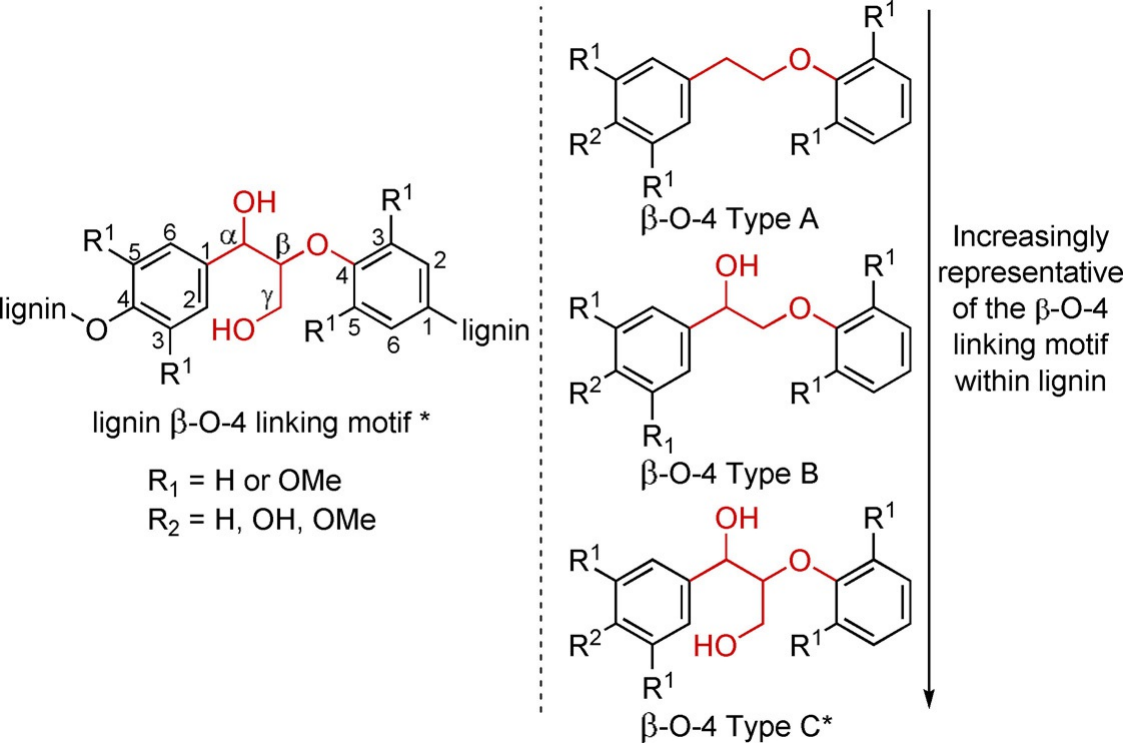
Native β‐O‐4 linking motif along with a series of β‐O‐4 model compound types, (β‐O‐4 Type A, β‐O‐4 Type B and β‐O‐4 Type C) ordered by how representative these structures represent the functional groups present in the native β‐O‐4 unit in lignin. *Note: These structures exist as diastereomeric mixtures.

The β‐O‐4 linking motif is the most abundant linking motif in native lignin (Figure 2) and is undoubtedly the most often replicated one in the literature. Consequently, a wide variety of model compounds, with differing levels of resemblance to the native β‐O‐4 motif in lignin, have been used to study this motifs’ reactivity. The simplest β‐O‐4 Type A model is (2‐phenoxyethyl)benzene, where R_1_=R_2_=H, is often used as a model compound as it is commercially available.^26^, ^27^, ^28^, ^29^, ^30^, ^31^, ^32^ Variations on β‐O‐4 Type A models with different substitution patterns on the aromatic rings can be readily synthesized via Williamson ether synthesis‐type reactions using (2‐bromoethyl)benzene derivatives containing the appropriate substituents on the aromatic ring with the desired phenol.^33^ β‐O‐4 Type A models, however, lack both the α and γ hydroxyl groups present in the native β‐O‐4 motif, which results in significantly different reactivity. Most studies have thus turned to β‐O‐4 Type B and β‐O‐4 Type C models, which incorporate the benzylic hydroxyl group at the α position. β‐O‐4 Type A and β‐O‐4 Type B models can be grouped as being C_6_–C_2_ compounds (C_6_ of the aromatic ring and the C_2_ of the ethyl chain) and are distinct from the C_6_–C_3_ β‐O‐4 Type C compounds, which incorporate the γ‐carbinol group (−CH_2_OH). β‐O‐4 Type C compounds are the most representative models of the β‐O‐4 linking motif. Also note that the inclusion of the γ carbon atom leads to the addition of a second stereocenter and thus a set of diastereomers (see below).

For β‐O‐4 Type A, B, and C models the methoxy group substitution patterns on the aromatic ring that mimic all combinations of H, G, and S monomer units (shown in Figure 1) have been prepared and used. Models that incorporate a phenolic group at R^2^ (Figure 2) are considered to represent β‐O‐4 linking motifs at the end of the lignin chain while in models where a methoxy group is incorporated at this position are considered to represent internal β‐O‐4 motifs.

**Scheme 1:**
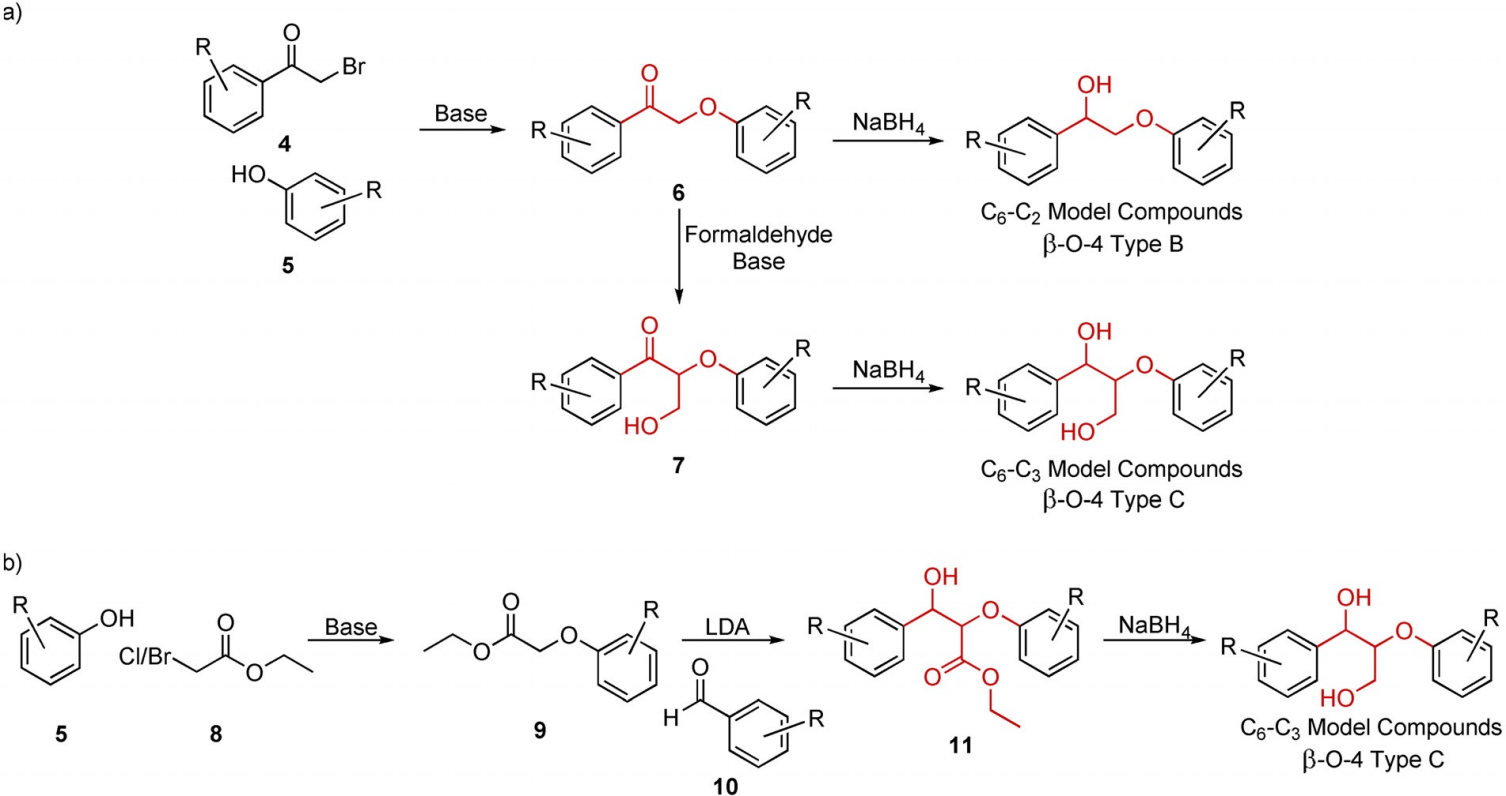
a) Generalized route to access β‐O‐4 Type B model compounds as well as β‐O‐4 Type C model compounds developed by Adler et al.^46^ b) Generalized route to accessing β‐O‐4 Type C model compounds developed by Nakatsubo et al.^55^ This route has been widely used and developed further by many researchers.^45^, ^50^, ^58^, ^80^

β‐O‐4 Type B model compounds are readily accessible in high yield via the synthetic route shown in Scheme 1 a. Coupling of a 2‐bromoacetophenone (**4**) with a phenol derivative (**5**) using a base (typically K_2_CO_3_, for example in acetone) generates the ketoether intermediate **6**, which is readily reduced using, typically, NaBH_4_ to obtain the β‐O‐4 Type B model compounds. Where the desired bromoacetophenone starting materials are not commercially available, they can be accessed from the parent acetophenone via bromination, for example, by reacting with Br_2_ in chloroform, ether, or ethanol followed by purification by recrystallization.^34^, ^35^, ^36^ The use of phenolic protecting groups such as benzyl (OBn)^34^, ^35^ or acetate^37^, ^38^, ^39^ on the acetophenone prior to bromination allows access to phenolic models. In these cases, the conditions used for the bromination should be chosen or modified accordingly; for example, N_2_ sparging (to remove HBr) can be beneficial when OBn groups are present^40^ whereas acetate protecting groups preclude the use of alcoholic solvents. Syringyl‐type acetophenones can be more challenging to selectively brominate than other analogues and therefore reagents such as CuBr_2_, pyridine (Py)⋅Br_3_ or 4‐dimethylaminopyridine (DMAP)⋅Br_3_ have been used as alternative brominating agents offering superior chemoselectivity.^41^, ^42^ Conveniently, compounds such as **6** and β‐O‐4 Type B models tend to be crystalline solids allowing for straightforward purification by recrystallization, enabling large‐laboratory‐scale synthesis by anyone with basic chemistry training and equipment.

**Figure 3:**
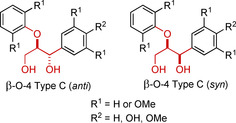
Generic structures of the *anti* and *syn* isomers of the β‐O‐4 Type C model compound.

An important consideration prior to discussing the synthesis of β‐O‐4 Type C model compounds is that these compounds contain two stereocenters, resulting in two diastereomers and four enantiomers of β‐O‐4 Type C model exist. The two diastereomers, *anti* (alternatively termed *erythro*) and *syn* (alternatively termed *threo*), are shown in Figure 3. In native lignin the ratio between the diastereomers is controlled by the selectivity of the addition of water to the quinone methide during the lignification process. In general, this has been shown to yield a ≈1:1 ratio of diastereomers in softwood lignins and closer to ≈3:1 in hardwood lignins, with S units favoring the formation of *anti* isomers.^16^, ^43^

The synthesis of diastereomerically pure and mixtures of diastereomers of β‐O‐4 Type C model compound have been developed (see below). A selective synthetic route to enantiomerically pure β‐O‐4 Type C model compounds has also been developed. This nine‐step route (not discussed in detail here) involving multiple protection/deprotection steps can be used to access the target compounds in moderate‐to‐good yields.^44^, ^45^

Adler et al. and later also others developed a methodology to access β‐O‐4 Type C model compounds from the intermediate **6** (Scheme 1 b, referred to as the Adler method henceforth).^46^, ^47^ This involves carrying out an aldol reaction between formaldehyde and **6** to generate **7**, using K_2_CO_3_ as a base. Today, 1,4‐dioxane is the most common solvent for performing this reaction and in our experience it is beneficial in limiting the formation of potential dehydration products. It should be noted, however, that the propensity of compounds to undergo dehydration appears to be highly substrate dependent. Recently, new conditions have been reported using catalytic amounts of KOH in 1,4‐dioxane/water giving improved yields with significantly reduced reaction times.^48^ Deuteration of the β‐position protons can be achieved by treating compounds such as **6** with K_2_CO_3_ in D_2_O, subsequent aldol reaction with formaldehyde using an [D_6_]acetone/EtOD solvent mixture resulted in a β‐deuterated compound **7**.^49^, ^50^ Such compounds can be very useful for mechanistic studies. The synthesis of β‐O‐4 Type C model compounds is completed by reduction of the ketone group to give **7**, typically using NaBH_4_. The choice of reducing agent as well as solvent selection during the reduction step has been shown to affect the diastereomeric ratios of the resultant β‐O‐4 Type C model compounds. The use of NaBH_4_ in 50:50 H_2_O/methanol can produce up to 86:20 *syn*/*anti* ratios while the use of *i*PrOH as solvent produces 36:64 *syn*/*anti*. For the production of more *anti*‐enriched products, LiAlH_4_ in THF can be used to achieve up to 25:75 *syn*/*anti*.^51^ Deuterium labeling of the α‐position can be achieved by replacing NaBH_4_ with NaBD_4_ and using a THF/D_2_O solvent system during the reduction step.^49^ Partly as a result of being mixtures of diastereomers, β‐O‐4 Type C models compounds are typically somewhat harder to purify and handle than β‐O‐4 Type B models as they are often obtained as sticky pastes or oils that occasionally crystallize on longtime standing after rigorous purification and drying. The Adler methodology is particularly valuable in the synthesis of models bearing the G–G type substitution pattern for both phenolic and non‐phenolic models.^52^ The ready availability and low cost of the required starting materials and the fact that all intermediate compounds can be purified by recrystallization means G–G, and to a lesser extent G–S, β‐O‐4 Type C models can be accessed on a multigram scale in a matter of days. Although less well suited to the large‐scale synthesis of S–H/G/S β‐O‐4 Type C models, this methodology remains exceptionally valuable for the synthesis of γ‐functionalized and more elaborate models. For example, γ‐acylated (e.g., *p*‐hydroxybenzoate, coumarate, ferulate, acetate) models are commonly synthesized via this method as well as tricin‐containing models.^53^, ^54^

A second commonly used route to β‐O‐4 Type C model compounds was developed by Nakasubo et al. (henceforth the Nakasubo method), outlined in Scheme 1 b.^55^ This route involves the generation of an aryloxyester such as **9** from the reaction of a chloro‐ or bromoacetate **8** (a potent lachrymator) with the desired phenol **5**. This can be achieved by reacting the two components in refluxing acetone with K_2_CO_3_, giving the desired ester in generally high‐to‐quantitative yields without the need for purification.^56^, ^57^, ^58^, ^59^ Compounds of the type **9** are then reacted with a benzaldehyde derivative **10** under aldol reaction conditions (−78 °C, lithium diisopropylamide, (LDA) in dry THF) to form the ester product **11**. Notably, this reaction can be carried out in one pot without needing to preform the ester enolate, as is commonly practiced,^58^ simplifying the reaction. G–G esters of type **11** can be purified by precipitation from diethyl ether in good yield; however, this is less efficient with S–S‐type esters, and column chromatography is usually required to achieve good yields. Reduction of the ester in **11** gives access to β‐O‐4 Type C model compounds; this can be achieved by using LiAlH_4_ or NaBH_4_.^45^, ^58^ Di‐γ‐deuterated β‐O‐4 Type C models can access by using NaBD_4_ (or LiAlD_4_) as the reducing agent.^60^, ^61^ As with the Adler method, phenolic models can be accessed by the integration of a benzyl‐protected group on the appropriate position of the starting material. The benzyl group can be readily removed by hydrogenolysis under mild conditions (Pd/C, 1 atm H_2_). The Nakasubo methodology produces β‐O‐4 Type C model compound mixtures of diastereomers. Ester aldol reactions have a transition state predetermined *anti* selectivity when the ester group employed is not sterically bulky^62^ (approximately 5:1 *anti*/*syn* ratios is observed when ethylesters are used).^45^ A development of the Nakasubo method employing sterically bulky esters such as *tert*‐butyl‐aryloxyesters was able to overcome this transition state predetermined *anti* selectivity as the steric bulk of the *tert*‐butyl‐esters made the transition state leading to the *syn* and the *anti* products more equal, allowing for a 1:1 *anti*/*syn* ratio to be achieved with some substrates.^45^ Prior to reduction, the *anti*/*syn* mixtures of **11** are often separable via column chromatography (this somewhat depends on the substitution pattern of the aromatic rings and the type of ester group used); indeed, Bolm and co‐workers reported the preparation of a range of diastereomerically pure β‐O‐4 Type C model compounds by using *tert*‐butyl‐aryloxyesters and subsequent careful silica gel chromatography.^45^ Alternatively, an *anti*‐enriched fraction of esters **11** can, in some cases, be recrystallized to give a pure *anti* product. Pure *syn* β‐O‐4 Type C model compounds have also been prepared via the hydroboration of (*Z*)‐α‐(2‐methoxyphenoxy),3,4‐dimethoxycinnamic acid, although yields throughout this synthesis are unfortunately poor.^63^, ^64^

The synthetic routes to β‐O‐4 Type B and C models outlined above cover the most frequently used methods; however, other less frequently used methodologies including, for example, an approach utilizing bromoketoesters as intermediates have also been developed. Details of these routes can be found elsewhere.^64^, ^65^, ^66^, ^67^, ^68^

The two main routes described here (Nakasubo and Adler methods) to access β‐O‐4 Type C model compounds have both advantages and disadvantages and are thus used intermittently between different research groups based on available equipment and materials, experience, and the type of desired substitution patterns on the aromatic rings. From a practical standpoint, the advantages of the Adler method are that it does not involve the use of particularly air‐ or moisture‐sensitive reagents and does not require the use of cryogenic temperatures as the Nakasubo method does. Therefore, a somewhat better‐equipped laboratory and a more highly trained chemist is required to carry out the synthesis via the Nakasubo method. The Adler method also has the advantage of having a point of divergence in the sequence, allowing access to β‐O‐4 Type B model compounds to which the Nakasubo method does not give access. The disadvantages of the Adler method are the lack of availability or prohibitive cost of the various acetophenone derivatives and the fact that the required bromination reaction can be troublesome with some substrates. Starting‐material availability is less of an issue with the Nakasubo method. The Nakasubo method produces good‐to‐excellent yields over a wide variety of substrates with little substituent effect issues being encountered. In our hands the Adler methods is preferred for the synthesis of basic G‐G β‐O‐4 Type C model compounds, while for other aromatic substitution patterns the Nakasubo method is preferred.

#### 2.1.2 Modified β‐O‐4 model compounds

The β‐O‐4 linking motif is often subjected to reaction conditions that result in alterations to its structure during lignin processing and is also frequently the target of selective modification strategies to either produce lignin with specific functionalities or that can facilitate depolymerization. Modification protocols for the model systems described above have been developed to assist in the study of these modified lignins and to facilitate reactivity and depolymerization studies. Below, we will discuss a few such examples that give modified model compounds in high yield.

**Scheme 2:**
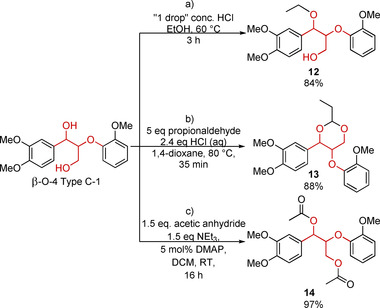
Examples of structurally modified β‐O‐4 Type C linking motif model compounds, a) α‐alkoxylated,^69^ b) α,γ‐diol protected,^75^ and c) acetylation.^77^ DCM=dichloromethane.

During extraction procedures aimed at retaining the core β‐O‐4 linking motif structure, protective modification is often carried out. Under acidic conditions in alcohol solvents the hydroxy group at the α‐position of the β‐O‐4 linking motif is readily converted to its corresponding ether, Scheme 2 a, sometimes noted as β‐O‐4‐αOR (see Figure 1) or β′‐O‐4. Model compounds with such a modification to the β‐O‐4 linking motif structure of both the β‐O‐4 Type B and C can be accessed via reaction under acidic conditions (cat. HCl) in the desired alcohol or ≈1:1 mixtures of 1,4‐dioxane and the desired alcohol at mild temperatures (60–80 °C). Moderate‐to‐high yields of the α‐alkoxylated product (65–84 %) can be obtained for linear alcohols, with ethanol, resulting in compound **12**, and butanol being the most commonly used.^69^, ^70^, ^71^, ^72^ A more recently developed protective modification approach developed by Luterbacher and co‐workers, uses aldehydes to form a cyclic acetal with the 1,3‐diol in the backbone of the β‐O‐4 linking motif. This approach reduces undesirable reactions such as linkage cleavage and/or repolymerization from occurring during lignin extraction. 1,3‐Diol‐protected model compounds can be accessed via reaction of a β‐O‐4 Type C model with HCl and an aldehyde of choice in 1,4‐dioxane as solvent at 80 °C, Scheme 2 b.^73^, ^74^, ^75^

A commonly encountered modification of lignin that has been applied to corresponding β‐O‐4 Type C model compounds is acetylation, Scheme 2 c. This modification is usually carried out to aid the solubility of lignin as it has been found that acetylation enhances lignin solubility in many organic solvents.^76^ Commonly used acetylation procedures for both lignin and model compounds alike utilize acetic anhydride and an amine base (pyridine or 1‐methylimidazole) reacting at room temperature for 16–24 h to produce the desired peracetylated products (e.g., **14**) in quantitative/near quantitative yields.^23^, ^24^, ^77^, ^78^

An important modification technique primarily targeted towards lignin degradation and functionalization is selective oxidation. This approach is based on an appreciation that oxidation of either the α or the γ alcohols of the β‐O‐4 linking motif results in a decrease in the bond dissociation energy of the C−O bond in the motif by ≈10 kcal mol^−1^ and opens up opportunities for new chemical transformations to be applied. This has resulted in a large number of approaches being developed to achieve selective oxidation.^79^

**Scheme 3:**
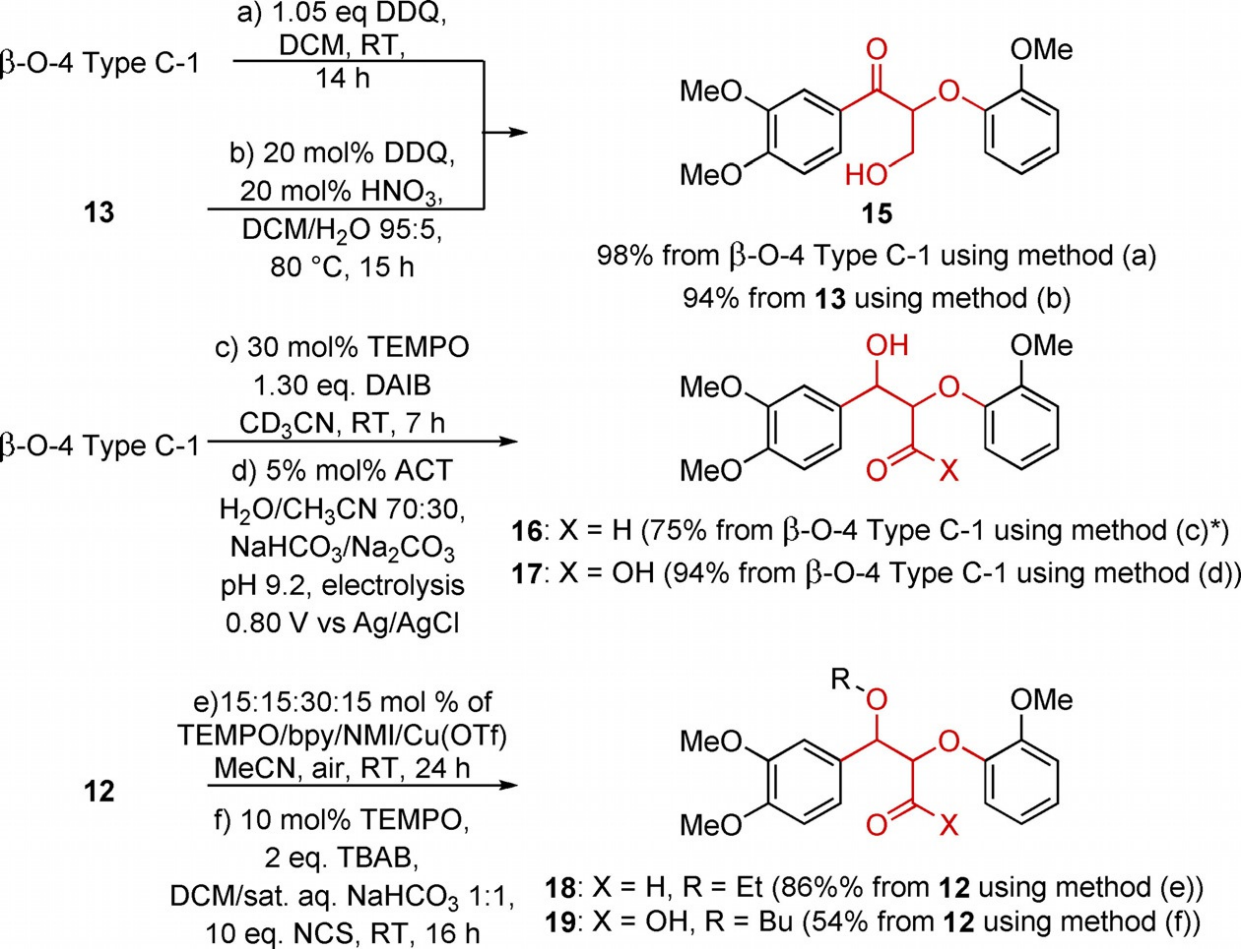
Examples of selective oxidative structural modifications of the β‐O‐4 Type C model compounds that are reported, starting from the α,γ‐diol, the α,γ‐diol protected and α‐etherified linkage structure. Full details of the procedures can be found in the related references: a) Ref. 58, b) Ref. 75, c) Ref. 82, d) Ref. 83, e) Ref. 72, and f) Ref. 84; *not isolated. 4‐ACT=acetamido‐TEMPO; bpy=2,2′‐bipyridine; NMI=1‐methylimidazole; TBAB=tetra‐*n*‐butylammonium bromide; NCS=*N*‐chlorosuccinimide.

Accessing benzylically oxidized β‐O‐4 Type C models is by far the most explored area; indeed, compounds of general structure **7** (Scheme 1 a) obtained as an intermediate during the Adler method gives direct access to benzylically oxidized β‐O‐4 Type C models. When starting from the β‐O‐4 Type C‐1 model stoichiometric approaches utilizing 2,3‐dichloro‐5,6‐dicyano‐1,4‐benzoquinone (DDQ)^58^ and 2,2,6,6‐tetramethylpiperidin‐1‐yl)oxyl (TEMPO) derivatives^80^ are often also quite convenient to achieve selective benzylic oxidation (Scheme 3). More elegant and green catalytic versions of these approaches that utilize molecular oxygen as the terminal oxidant have also been used.^58^, ^80^ Photocatalytic and mechanochemical approaches have also been developed for this transformation.^57^, ^81^ A modification of the catalytic DDQ approach has also been developed to facilitate the benzylic oxidation of the α,γ‐diol‐protected β‐O‐4 linking motif such as compound **13**.^75^

The β‐O‐4 Type C‐1 model has also successfully been converted via primary oxidation to its aldehyde **16** using a selective TEMPO/(diacetoxyiodo)benzene (DAIB) approach^82^ or to its carboxylic acid derivative **17** employing a 4‐acetamido–TEMPO‐mediated electrochemical procedure.^83^ Alternatively, methods for the production of aldehyde **18** or carboxylic acid derivatives **19** of α‐etherified β‐O‐4 Type C models such as **12** have been developed.^70^, ^72^, ^84^ The benzylic alkoxy group prevents degradation via a retro‐aldol pathway and thus improves the stability of **18** in particular.

### 2.2 β‐5 Type model compounds

**Figure 4:**
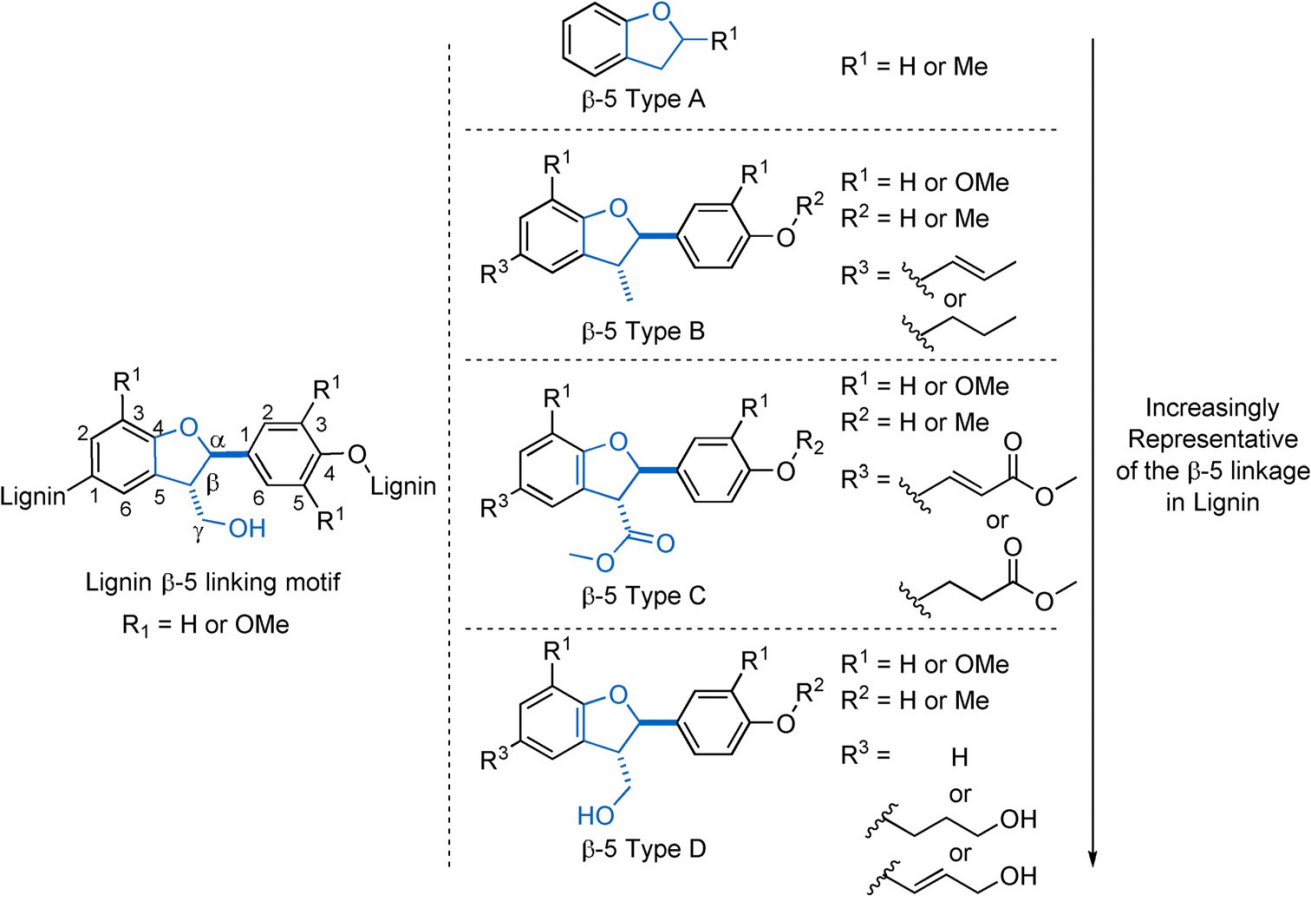
Native β‐5 linking motif along with a series of model compounds of the β‐5 linking motif that have been used to study its structure and the reactivity.

The β‐5 linking motif is one of the primary linking motifs in lignin, making up 9–12 % of high‐G‐content lignins. Due to the lower abundance of this linking motif compared to the β‐O‐4 linking motif, the use of model compounds for studying its chemistry has been less well developed. Nevertheless, many examples of model compounds of the β‐5 linking motif can be found in the literature of varying levels of complexity and resemblance to the native structure as outlined in Figure 4. The relative stereochemistry of the β‐5 linking motif has been shown to be *trans* (Figure 4), with *cis* being present in negligible quantities, if at all.^43^ A computational study utilizing model substrates was used to determine that this stereochemistry is derived from the ring‐closing reaction following the radical dimerization which forms the β‐5 bond. This ring closing is believed to be under thermodynamic rather than kinetic control, allowing the more stable *trans* relationship of the substituents to form.^85^ Thus, typically, β‐5 linking motif model compounds are synthesized and used in the *trans* form. 2,3‐Dihydrobenzofuran and its 2‐methyl derivative (β‐5‐Type A) are the simplest model systems of the β‐5 linking motif. This types of model compounds lack most of the functionality present in lignin but are cheap, commercially available compounds. Therefore, β‐5‐Type A model compounds are often utilized, even as general models to represent aromatic–aliphatic ether linking motifs found in lignin.^86^, ^87^ Two main approaches have been taken to achieve the synthesis of the more complex β‐5 model compounds (β‐5‐Types B, C, and D). These are oxidative phenol coupling (β‐5 Method 1), utilizing either a metal (β‐5 Method 1 a) or an enzyme (β‐5 Method 1 b) to carry out the required single‐electron oxidation or an acid‐catalyzed rearrangement of chalcone epoxides (β‐5 Method 2). Both approaches will be outlined in more detail below.

**Scheme 4:**
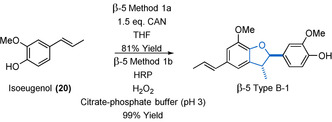
Chemical (β‐5 Method 1 a) and enzymatic (β‐5 Method 1 b) approaches to the synthesis of the β‐5‐linked isoeugenol dimer β‐5 Type B‐1.^93^, ^95^

The type of models that can be obtained via oxidative phenol coupling (β‐5 Method 1) depend on the starting material used. Models of the β‐5‐Type B can be synthesized via the radical dimerization of isoeugenol (**20**) (Scheme 4). First reported as early as the 1900s, this reaction has been developed in subsequent decades and used by many researchers.^88^, ^89^, ^90^, ^91^, ^92^, ^93^ A common β‐5 Method 1 a approach is to use a single‐electron oxidant such as FeCl_3_. As an alternative, ceric ammonium nitrate (CAN) has recently been reported to produce better yields (30 % yield using FeCl_3_, 81 % using CAN).^91^, ^93^ Enzymatic methodologies (β‐5 Method 1 b) have been developed for this reaction, initially using oxygen‐laccase enzymes.^88^, ^94^ Subsequently, horseradish peroxidase (HRP) enzymes have been found to be excellent catalysts for this transformation with yields of 99 % being achieved.^94^, ^95^, ^96^ Methylation of the phenolic compound β‐5 Type B‐1 can be used to access its non‐phenolic analogues in high yield via standard phenol methylation procedures.^93^, ^97^ Pd/C reductions of the alkene in β‐5 Type B‐1 or its methylated derivative under H_2_ can give access to their propyl chain‐containing analogues (Figure 4, β‐5 Type B models R^3^=propyl).^98^

An advantage of β‐5 Type B model compounds is that they can be accessed in just a few steps, with each one giving good‐to‐excellent yields. There are, however, significant drawbacks to the use of β‐5 Type B models. The lack of any functionality on the γ‐carbon atom of the β‐5 core leads to significantly different reactivity compared to the native β‐5 linking motif. In this respect, β‐5 Type C (containing esters) and β‐5 Type D (containing the native hydroxyl) model compounds are an improvement as they incorporate functionality at the γ position.

**Scheme 5:**
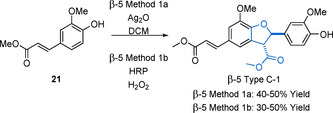
Chemical and enzymatic approaches to the synthesis of the β‐5 Type C‐1, and example of a β‐5 linked ferulate ester dimer.

Ferulate ester dimerization gives access to β‐5 Type C model compounds that contain additional ester groups at the γ‐positions when compared with the β‐5 Type B models (Scheme 5). The approach to the synthesis of these compounds is similar to that of the isoeugenol dimers described above, with both β‐5 Method 1 a, (chemical)^85^, ^99^, ^100^, ^101^ and β‐5 Method 1 b (enzymatic)^102^, ^103^, ^104^, ^105^ dimerization procedures being employed.

The best results in accessing model compounds of the β‐5 Type C‐1 is β‐5 Method 1 a using Ag_2_O. Reactions carried out in a mixed acetone–benzene solvent system produce yields of 40 %.^106^ Other, more practical, solvents such as DCM have been employed giving similar yields.^85^ In our experience, the best yields from this reaction, which must be carried out in the absence of light, are obtained with very dry and degassed solvents, making it a challenge to scale up. Enzymatic dimerization (β‐5 Method 1 b) using HRP has proved quite easily scalable as it is carried out in aqueous conditions and has been used frequently to carry out this conversion with yields in the range of 30–50 %.^103^, ^104^, ^105^, ^107^ From a practical perspective, β‐5 Method 1 b has significant advantages over β‐5 Method 1 a; that is, less use of organic solvents, no necessity to go through extensive drying and degassing procedures, the generation of less waste, and ease of scale‐up lead to a preference for the use of this method. Access to β‐5 models with S–S substitution patterns is not possible due to the lack of a free 5‐position on the aromatic ring. However, β‐5 Type C models with H–H substitution patterns have been accessed via the general methods β‐5 Method 1 a^108^, ^109^ and β‐5 Method 1 b^110^ using methyl *p*‐hydroxycinnamate as starting material. The synthesis of mixed G–S models has also been accomplished using, for example, β‐5 Method 1 b and a mixture of methyl ferulate (**21**) and methyl sinapate. This method, however, suffers from poor yields of the desired product (24 %) due to the competing consumption of the starting materials in homodimerization reactions.^111^

**Scheme 6:**
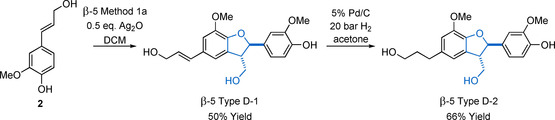
Synthesis of compound β‐5 Type D‐1 and β‐5 Type D‐2 from **2**.

As with β‐5 Type B models, β‐5 Type C models can be methylated to access their non‐phenolic analogues using methyl iodide and a base; however, β‐5 Type C compounds are susceptible to ring‐opening reactions under basic conditions.^56^ In both phenolic and non‐phenolic models, the double bond can be readily reduced under standard conditions with Pd/C.^100^ Access to β‐5 Type D‐1 and β‐5 Type D‐2 models can be achieved via LiAlH_4_ or diisobutylaluminium hydride (DIBAL‐H) reduction of the ester groups of the appropriate β‐5 Type C models.^100^, ^112^ An alternative route to these β‐5 Type D models is to start from coniferyl alcohol, which can also undergo β‐5 Method 1 a dimerization with Ag_2_O to give compound β‐5 Type D‐1 directly (Scheme 6) in up to 50 % yield. Hydrogenation of the double bond then gives access to compound β‐5 Type D‐2.^24^, ^113^ Coniferyl alcohol is much more expensive than ferulic acid and therefore the ferulate‐based methods are usually preferred, especially for larger‐scale preparations.

The presence of functional groups on the propyl sidechain of the 2,3‐dihydrobenzofuran ring of the β‐5 linking motif can be of great significance in their usefulness as model substrates. An example of this can be seen in the use of the non‐phenolic derivative of the β‐5 Type D‐2 model. In the study of lignin acidolysis, the side chain proved to be inert to the reaction conditions and so the study of the reactivity of the β‐5 core was not complicated by side reactions.^56^ However, in the study of lignin oxidation, the sidechain proved to be reactive under the conditions being studied, complicating the study of the β‐5 core.^114^ This highlights the importance of choosing the correct model system and giving due consideration to side chains and their potential as complicating factors.

**Scheme 7:**
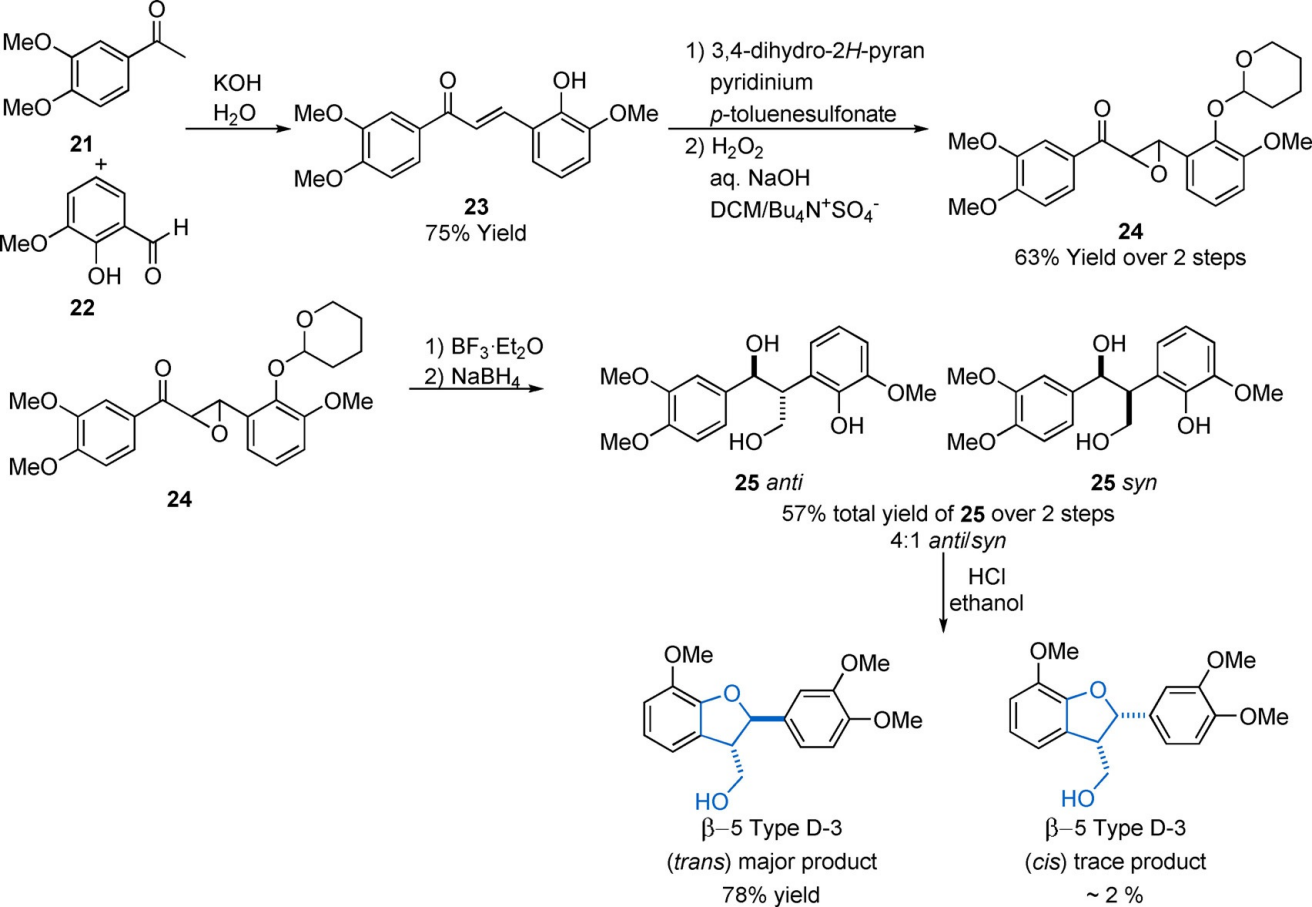
Synthetic route towards β‐5 type model compounds (β‐5 Method 2) developed by Lundquist and co‐workers.^115^, ^116^

An alternative route (β‐5 Method 2) for the synthesis of β‐5 Type D lignin model compounds where R_3_=H has been reported in the literature and is shown in Scheme 7. This methodology has the advantage of being able to provide access to β‐5 Type D‐3 model compounds, which have no side chain on the 2,3‐dihydrobenzofuran ring.^115^, ^116^ This route was initially reported by Brunow and Lundquist^115^ and was subsequently further developed.^116^ A Claisen–Schmidt condensation between an acetophenone derivative **21** and a phenolic benzaldehyde **22** is used to form the intermediate **23**. The phenol group in **23** is then protected prior to epoxidation to the chalcone epoxide **24**. Lewis acid‐catalyzed rearrangement of the chalcone epoxide leads to a diastereomeric mixture of **25** *anti* and **25** *cis*. Treatment of **25** with HCl, forms the desired *trans* β‐5 model compound as the major product. The *syn* product is also formed but only in small quantities (≈2 %). This is a versatile methodology that can also be applied to the synthesis of the phenolic analogue of β‐5 Type D‐3; however, this approach is rarely used due to the number of synthetic steps involved when compared to the single‐step dimerization procedure discussed previously in this section.^117^

### 2.3 β‐β‐Type model compounds

**Figure 5:**
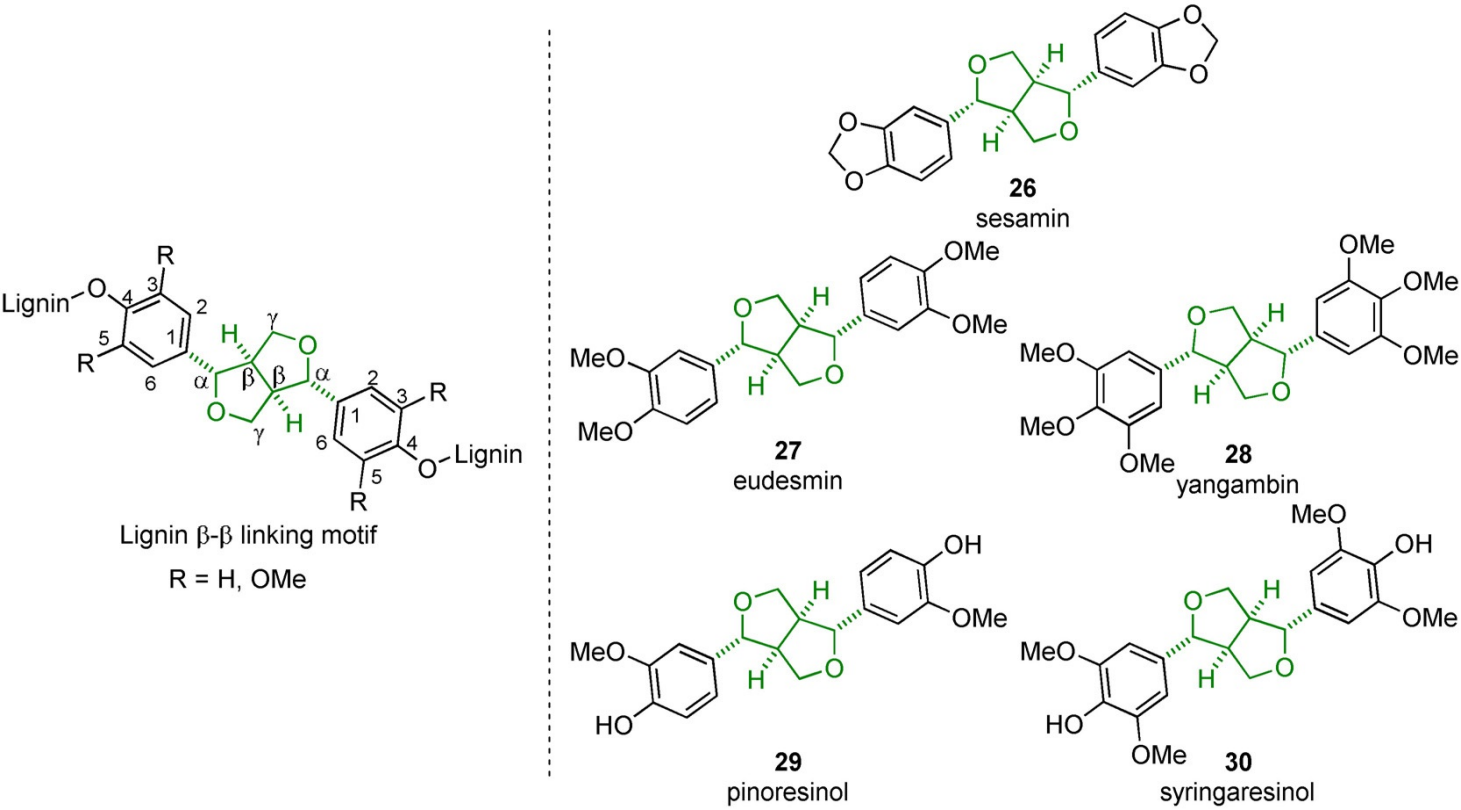
Native β‐β linking motif along with an array of β‐β lignin model compounds used to study its structure and the reactivity.

The β‐β linking motif (Figure 5) is unusual as during lignification *in planta* it can only form via monolignol dimerization reactions rather than through chain elongation. As shown in Table 1, the β‐β linking motif is found to make up between 3–12 % of the linking motifs in lignin. A series of model compounds that is used to study this linking motif is shown in Figure 5 and these compounds are obtained either synthetically or by extraction from natural sources.

Several approaches have been taken to synthesize the core unit of the β‐β linking motif as this type of compounds is also of interest for its potential biological properties, including antitumor, antiviral, immunosuppressant, and anti‐inflammatory effects.^118^ However, typically, many of these syntheses focus on obtaining isomers of the core unit with different configuration of the benzylic carbon atoms compared to the native β‐β lignin unit shown in Figure 5.^16^, ^119^ Therefore, only model compounds with the matching stereochemistry are discussed. Nonetheless, treatment of lignin during the extraction process or during degradation procedures (acidolysis for example) can result in the epimerization of the benzylic carbon atoms of the β‐β linking motif, resulting in different relative configurations.^56^

**Scheme 8:**
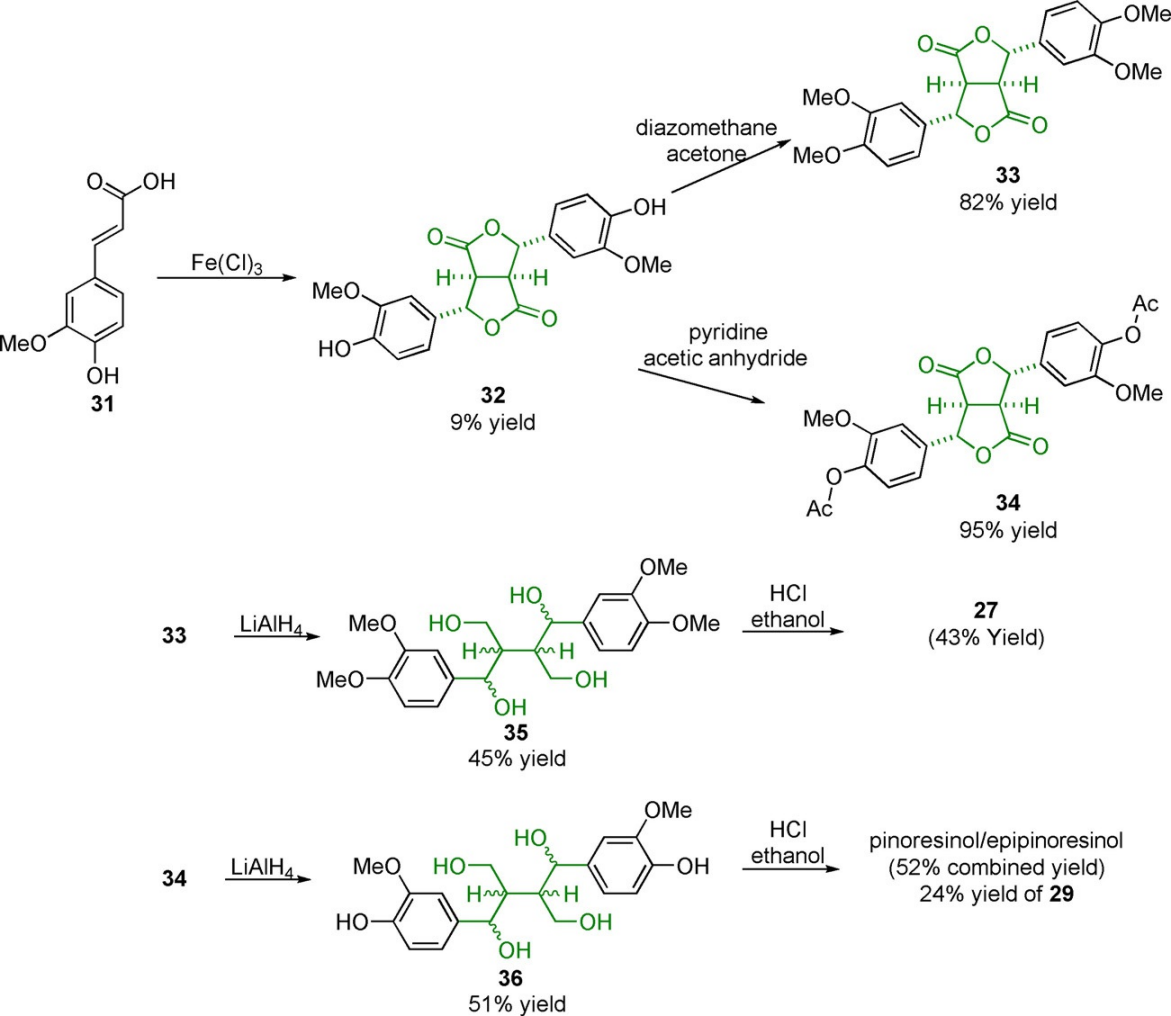
Synthesis of eudesmin (**27**) and pinoresinol (**29**) from ferulic acid (**31**).^123^.

Sesamin (**26**) is often a useful model compound for studying the softwood β‐β linking motif as it is found in sesame oil in 0.1–0.5 wt % and can be readily isolated through, for example, column chromatography.^56^, ^120^, ^121^, ^122^ The downside of sesamin as a model compound is that it contains a methylenedioxy group on both aromatic rings: a motif not found in native lignin. Eudesmin (**27**) and yangambin (**28**) can be considered as G–G (softwood) and S–S (hardwood) “internal” models where the phenols are connected to the rest of the lignin polymer chain. Chemical biomimetic dimerization is a popular approach to the formation of the β‐β linking motif as it allows for the construction of the complex core in a simple one‐step reaction. An interesting but not very practical synthesis reported in 1982, starting from ferulic acid (**31**), utilized a dimerization reaction using iron(III) chloride to produce the dilactone **32** (Scheme 8).^123^ This dilactone could be methylated to produce **33** or acetylated to produce **34**. LiAlH_4_ reduction of **33** and **34** gave the tetraols **35** and **36**, respectively. Acidic treatment of these tetraol compounds yielded the desired eudesmin (43 % yield) from compound **33** and pinoresinol (24 % yield) from compound **34**. This synthesis strategy is relatively long and suffers from an extremely low‐yielding initial dimerization step.

**Scheme 9:**
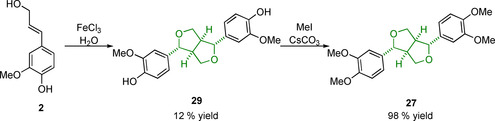
Example of a recent synthesis of pinoresinol (**29**) and eudesmin (**27**) from coniferyl alcohol (**2**).

Dimerization starting from coniferyl or sinapyl alcohol as opposed to their carboxylic acid derivatives was initially investigated in the 1950s by Freudenberg and Hübner^124^ and has since been further developed.^125^ This approach simplifies the route as the resinol structure is formed directly and so access to the desired phenolic pinoresinol/syringoresinol structures is achieved in one step. The reactions are, however, low yielding when pinoresinol structures are targeted. A simple methylation step can be employed to access the eudesmin/yangambin structures (Scheme 9).^126^

**Scheme 10:**
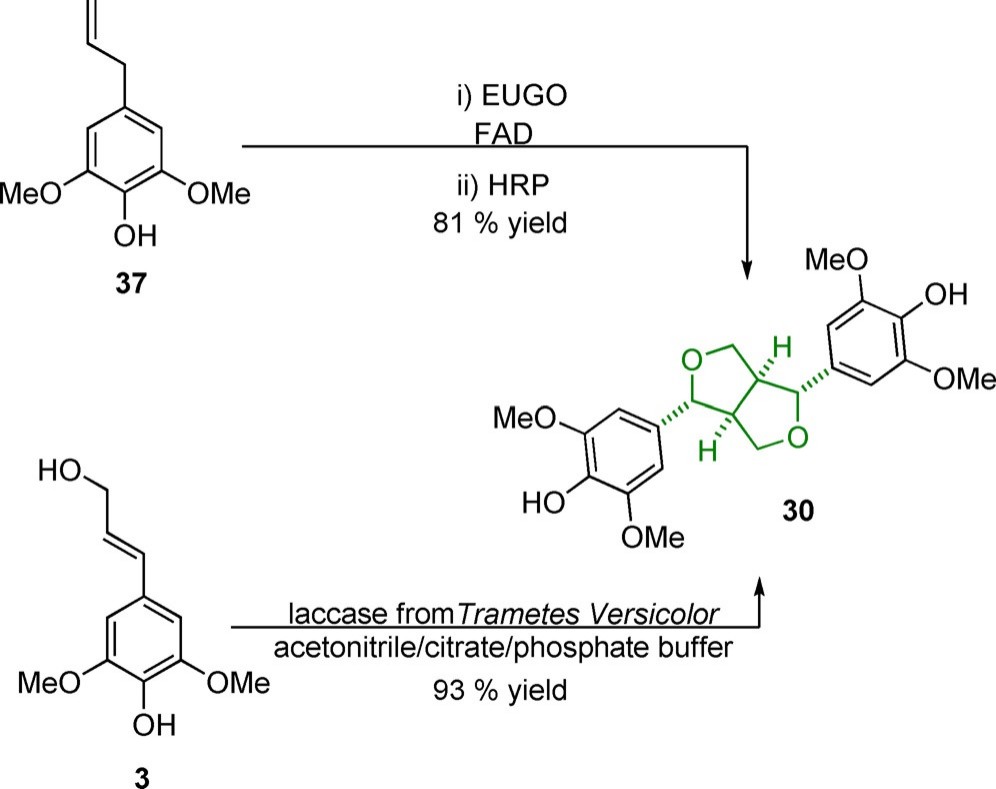
Synthetic routes used to access the β‐β linking motif model syringaresinol (**30**) from 2,6‐dimethoxy‐4‐allylphenol (**37**) and sinapyl alcohol (**3**). FAD=flavin adenine dinucleotide.

The synthesis of β‐β linking motif model compounds containing syringyl‐type aromatic groups are higher yielding than those containing the guaiacyl ones due, in part, to the 5‐position being “protected” against radical coupling reactions. Syringaresinol (**30**) can be synthesized starting from sinapyl alcohol (**3**) chemically using stoichiometric copper(II) sulfate in the presence of light and air with yields of 67 % being obtained following purification by crystallization.^127^ Enzymatic dimerization can be carried out starting from **3** using a laccase from *Trametes versicolor*, giving 93 % yield, or from the substantially cheaper 2,6‐dimethoxy‐4‐allylphenol (**37**) in a one‐pot two‐enzyme conversion (Scheme 10).^128^ The latter route involves the conversion of **31** initially to sinapyl alcohol via an eugenol oxidase (EUGO), a reaction that generates hydrogen peroxide; this hydrogen peroxide is then consumed by HRP in the dimerization of **3** to **30**, giving an 81 % yield over the two steps.^129^

**Scheme 11:**
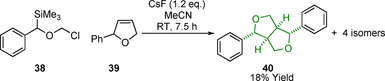
3+2 cycloaddition approach to the synthesis of resinol structures.

Some alternative approaches that do not involve dimerization have been used for the synthesis of resinol structures.^130^, ^131^ In the example shown in Scheme 11 a methodology utilizing Si‐based carbonyl ylides (**38**) is employed. The ylide reacts with an appropriate alkene **39** via a 3+2 cycloaddition, yielding compound **40**, which contains the β‐β linking motif core structure. The yield of **40** is, however, low at 18 % and is formed along with the other potential isomers.^131^ This low yield limits the widespread application of this methodology but potentially enables a synthetic route to access specific asymmetrical β‐β model compounds.

### 2.4 5‐5/Dibenzodioxocin‐type model compounds

**Figure 6:**
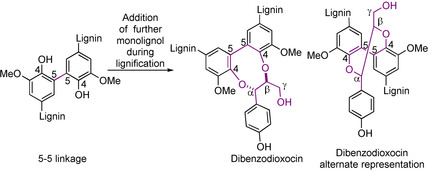
Structures of the 5‐5 linking motif as well as the native dibenzodioxocin linking motif.

**Scheme 12:**
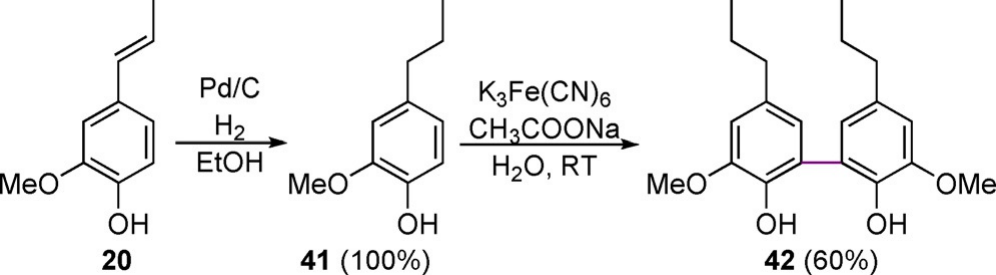
Synthesis of the 5‐5 linking motif core of the dibenzodioxocin model compound.

The 5‐5 linking motif in lignin makes up approximately 5–7 % of the total linking motifs.^3^ It has been shown that “essentially all” 5‐5 linking motifs in lignin are actually found as part of the dibenzodioxocin linking motif (Figure 6).^3^ The 5‐5 linking motif can be modelled using biphenyl‐type compounds, which are generally synthesized via dimerization reactions using sodium or potassium persulfate/iron(II) sulfate mixtures^91^, ^132^ or K_3_Fe(CN)_6_ 133, 134 as shown in Scheme 12. Alternatively, commercially available biphenyl or 2,2′ biphenol are often used.

**Figure 7:**
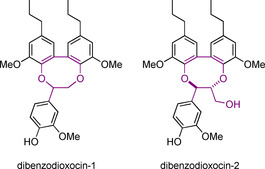
Model compounds of the dibenzodioxocin linking motif.

The synthesis of dibenzodioxocin models is relatively underexplored compared to β‐O‐4, β‐5, and β‐β models.^133^, ^135^‐^137^ There are, however, two reported synthetic routes that can be used to access these structures. The structures of dibenzodioxocin model compounds that can be accessed through these two routes are shown in Figure 7. The synthesis of both starts with the formation of a 5‐5 bond (Scheme 12). This first unit **42** is synthesized via the radical coupling of **41**, which itself is synthesized from isoeugenol **20**, mediated by K_3_Fe(CN)_6_.

**Scheme 13:**
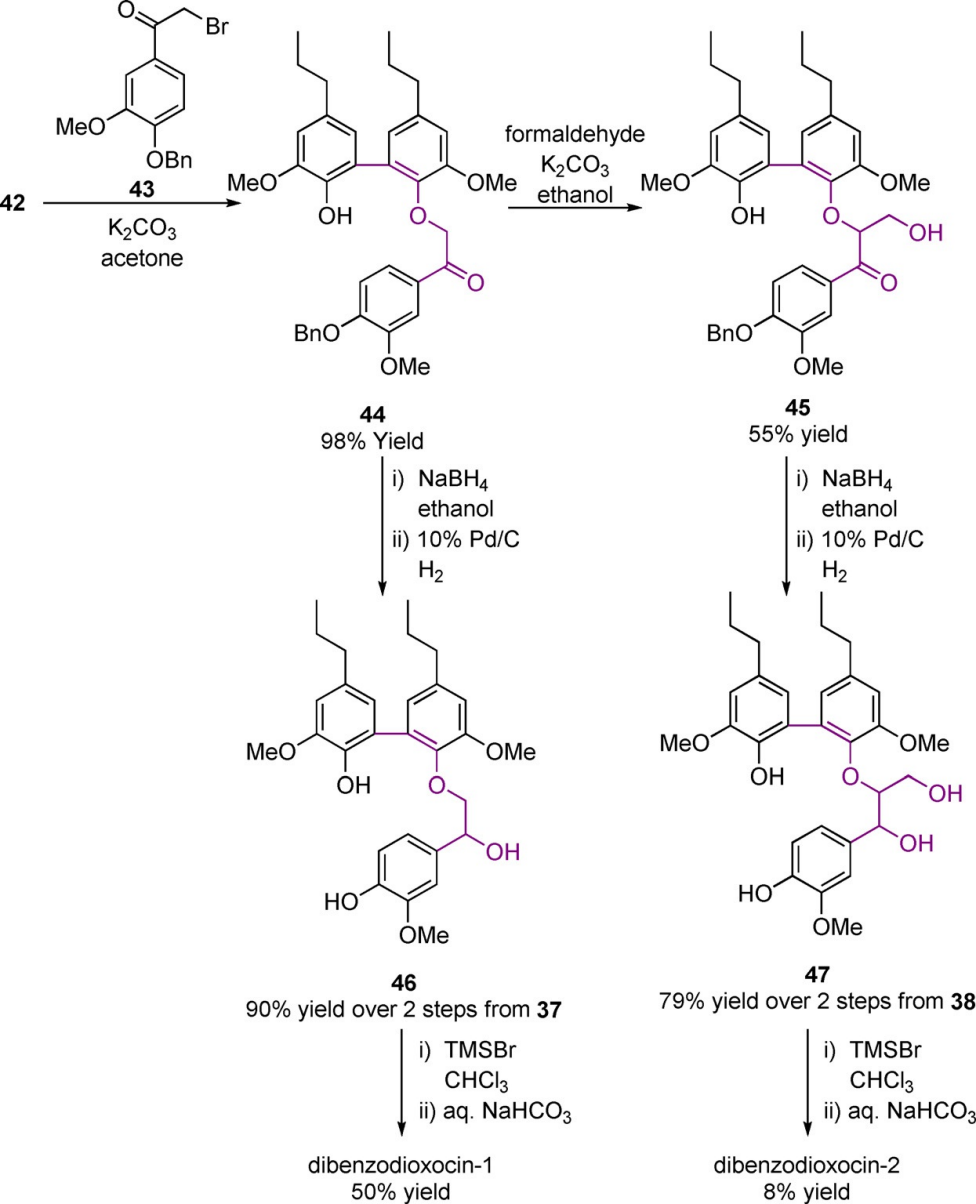
Synthetic route to the model compounds dibenzodioxocin‐1 and dibenzodioxocin‐2 via a stepwise approach starting from **42**.^133^, ^135^, ^136^, ^138^

The route from the 5‐5‐linked dimer to the dibenzodioxocin model described in Scheme 13 uses a 2‐bromoacetophenone derivative (**43**) to form the β‐aryl ether **44**. The model with the γ‐carbinol incorporated (dibenzodioxocin‐2) can be accessed via the use of the Adler method using formaldehyde with base to make β‐O‐4 Type C linking motifs, as shown in Scheme 1 a.^46^ In this case intermediate **45** is formed. Ketone reduction of either **44** or **45** followed by a benzyl deprotection step gives the free phenol intermediates **46** and **47**. An intramolecular cyclization reaction is initiated using trimethylsilyl bromide (TMSBr) to form a benzylic bromide. Aqueous NaHCO_3_ then generates a quinone methide to which the phenol adds to give the desired products. When this synthetic route is used to access dibenzodioxocin‐1, it gives a 50 % yield. However, when accessing dibenzodioxocin‐2, this route suffers from low yield (8%) due to the extremely low‐yielding final ring‐closing step.^135^, ^138^ There is probably still room for improvement with regard to this final step as previous work did not seem to have carried out further optimization. A phenol methylation procedure has been developed for dibenzodioxocin‐1 to study an etherified model of the dibenzodioxocin linking motif.^136^

**Scheme 14:**
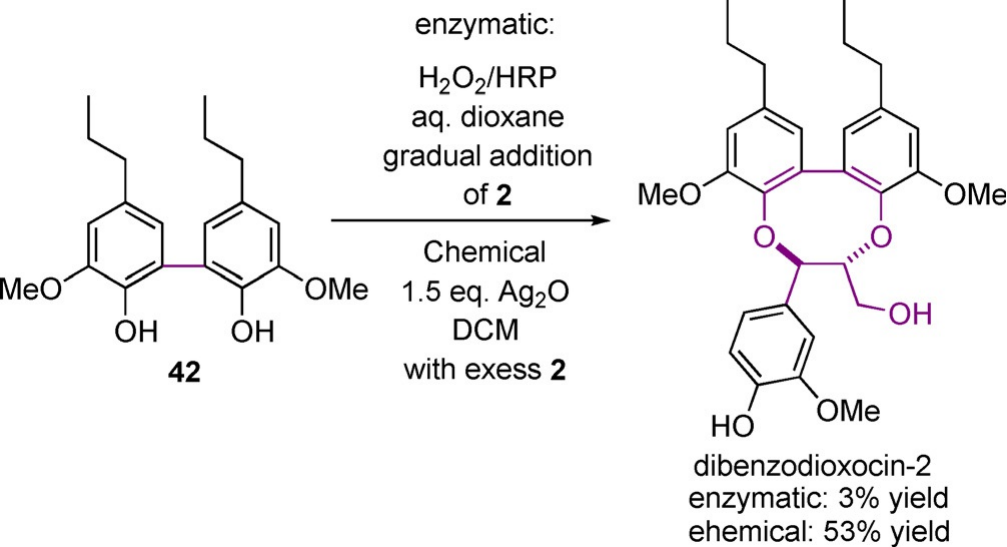
Radical oxidative approaches to the synthesis of the dibenzodioxocin model compound via enzymatic and chemical means.^135^, ^139^

An oxidative coupling approach to form the key dibenzodioxicin ring in dibenzodioxocin‐2 has proved more successful (Scheme 14). In one step, **42** is oxidatively coupled with **2** using Ag_2_O, giving dibenzodioxocin‐2 in reasonable 53 % yield. The alternative HRP/hydrogen peroxide‐mediated coupling, however, gave only a 3 % yield. Due to its low number of synthetic steps and relatively high yield, this final approach is the one that has been applied for producing dibenzodioxocin model compound dibenzodioxocin‐2 for use in reactivity studies.^133^

### 2.5 4‐O‐5‐type model compounds.

**Figure 8:**
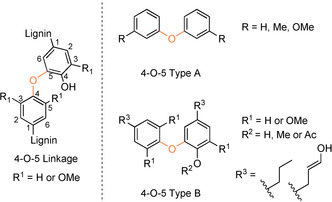
Native 4‐O‐5 linking motif along with the generalized structures of some of the model compounds that have been used to study the linking motif in isolation.

The 4‐O‐5 linking motif is not formed in the initial stages of the lignification process but rather via the coupling of lignin oligomers or dimers.^23^ As a minor motif it constitutes approximately 2 % of the linking motifs in lignin.^3^ Model compounds that have been used to study the 4‐O‐5 motif range from the widely used simple and commercially available diphenyl ether^140^, ^141^, ^142^ to substituted diphenyl ethers synthesized via Cu‐catalyzed arylation of phenols (**49**) with aryl halides (**48**),^140^, ^143^ to products of radical dimerization reactions.^107^, ^144^, ^145^ Examples of the models that have been used are shown in Figure 8. Linking motif models of the type 4‐O‐5 Type A contain ether‐linked aryl groups; however, these lack the aromatic substitution patterns seen in lignin. Linking motif models of the type 4‐O‐5 Type B with lignin‐like substitution patterns on the aromatic rings offer a closer match to the native 4‐O‐5 linkage.

**Scheme 15:**
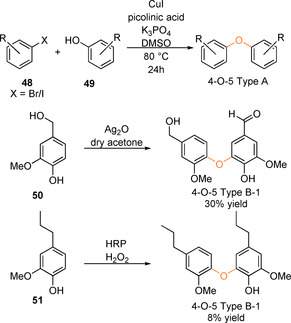
Examples of approaches taken towards the synthesis of 4‐O‐5 linking motif‐type model compounds.

4‐O‐5 Type B model synthesis is generally carried out via Ag_2_O‐mediated or peroxidase‐catalyzed radical dimerization reactions (Scheme 15). Selectivity towards 4‐O‐5‐coupled products and the prevention of oligomer formation are the primary issues in these reactions. The direct oxidation of vanillin with Ag_2_O results in a complex product mixture of oligomeric and polymeric products.^144^ However, when vanillyl alcohol (**50**) is used a 4‐O‐5 dimer is produced in which one of the alcohol groups is oxidized to the aldehyde. This is thought to occur via the formation of vanillin in situ, which then couples selectively at the 5‐positon with the 4‐O radical of vanillyl alcohol, producing the mixed dimer, 4‐O‐5 Type B‐1, in 30 % yield.^144^ This product is particularly useful as it can be modified through subsequent reactions to produce further derivatives for analysis of natural lignins.^22^, ^144^ Alternatively, 4‐O‐5 Type B‐1 has been used as a starting material for the production of more complex model systems (see Scheme 19). Enzymatic strategies such as the use of peroxidase enzymes for the 4‐O‐5 dimerization of 4‐propyl guaiacol (**51**) have been reported but suffer from poor yields. The dimerization of 4‐propyl guaiacol provides the 4‐O‐5 dimer as the minor component (8 %) in the product mixture whereas the 5‐5‐linked primary product **42** is obtained in a 56 % yield, as discussed in the previous section.^107^ A similar enzymatic dimerization reaction has been reported with a phenolic G–G β‐O‐4 Type C, giving only 2.9 % of the 4‐O‐5 coupled dimer.^23^

## 3 Multi‐Linking Motif Lignin Model Compounds

Numerous higher‐order lignin model compounds have been synthesized, made up of combinations of different linking motifs. Here, such models are classified as containing between 2 and (approximately) 7 linking motifs in a defined order. These can be relatively well characterized but still consist of complex mixtures of stereoisomers. The nomenclature for these multi‐linking motif compounds generally refers to the number of aromatic units that are in the oligomer rather than the number of linking motifs. Two synthetic strategies can be distinguished: 1) stepwise addition of linking motifs and 2) oxidative coupling reactions, which are separately discussed below.

### 3.1 Stepwise synthetic approaches

**Scheme 16:**
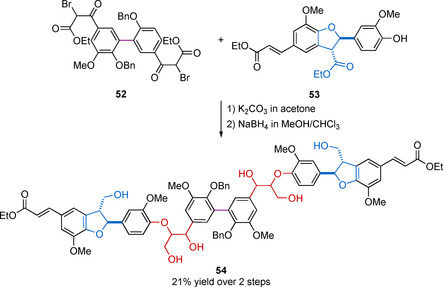
Example of a hexameric lignin model compound ((β**‐**5) (β‐O‐4) (5‐5) (β‐O‐4) (β**‐**5)), synthesized by Forsythe et al.^91^

Several stepwise approaches begin with the synthesis of a difunctional, often a symmetric 5‐5 or a 4‐O‐5 motif, which can then be extended to a sequentially symmetrical oligomer. This approach has been used to access tetramers^132^, ^146^ (3 linking motifs, (β‐O‐4) (5‐5) (β‐O‐4) as well as (β‐O‐4) (4‐O‐5) (β‐O‐4)), hexamers^91^ (5 linking motifs, (β**‐**5) (β‐O‐4) (5‐5) (β‐O‐4) (β**‐**5)), and octamers^91^ (7 linking motifs, (β**‐**5) (β‐O‐4) (β‐O‐4) (5‐5) (β‐O‐4) (β‐O‐4) (β**‐**5)). An excellent example of this approach from Forsythe et al. demonstrates the synthesis of a hexameric model (Scheme 16).^91^ Compound **52** containing a 5‐5 linking motif was initially synthesized from acetovanillone over four steps in 35 % yield. Compound **53** was synthesized using the β‐5 Method 1 a, as outlined in Scheme 5, starting from ethyl ferulate. The reaction between **52** and two equivalents of **53** under mildly basic conditions generated a hexameric intermediate. Chemoselective reduction with NaBH_4_ yielded **54** containing the β‐O‐4 and β‐5 linking motifs while retaining the cinnamate ester sidechains.

**Scheme 17:**
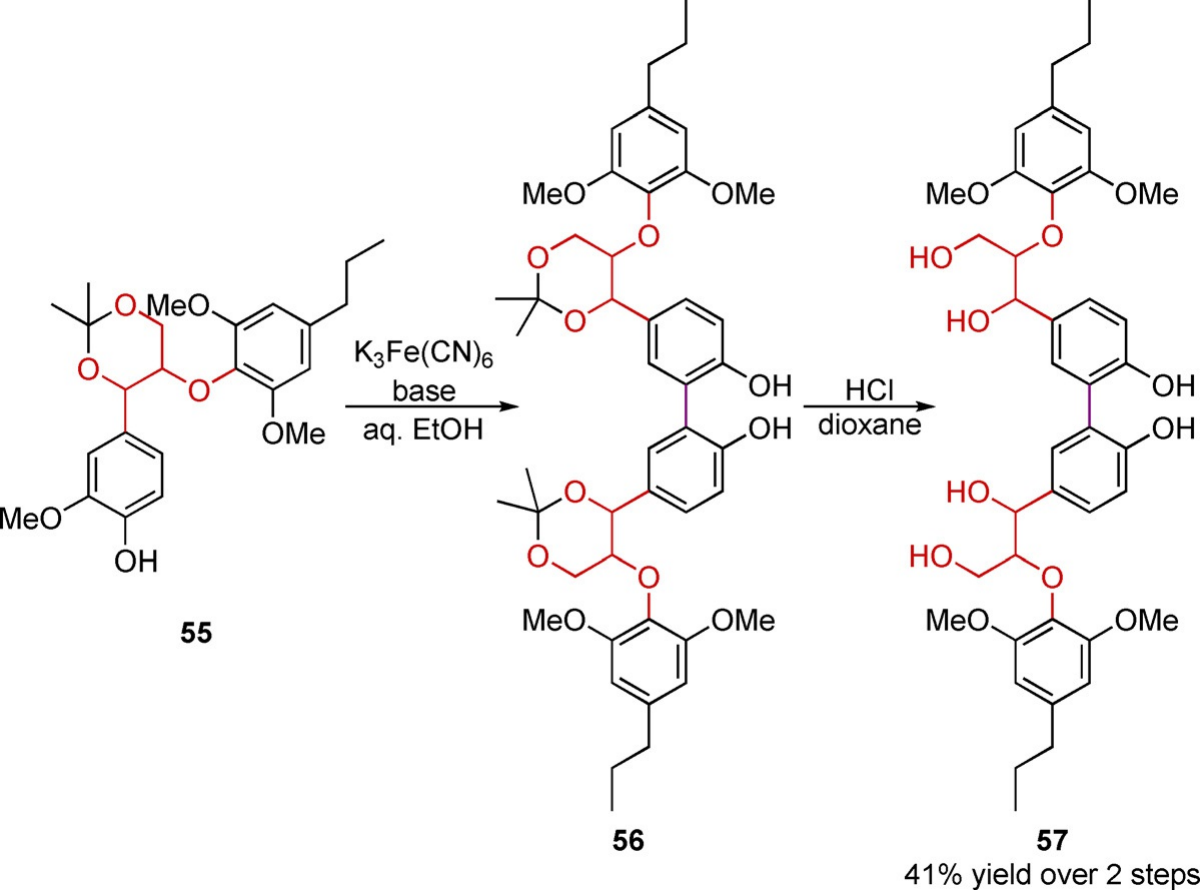
Example of a tetramer lignin model compound ((β‐O‐4) (5‐5) (β‐O‐4)) synthesized via a radical dimerization reaction.^147^, ^148^

A similar approach is to synthesize dimeric or trimeric sequences of linking motifs that are then subjected to radical dimerization reactions. This has been used to generate tetramers^147^, ^148^ with three linking motifs—(β‐O‐4) (5‐5) (β‐O‐4) and (β**‐**5) (5‐5) (β**‐**5)—and hexamers^149^ with 5 linking motifs—(β‐O‐4) (β‐O‐4) (5‐5) (β‐O‐4) (β‐O‐4). An example of how this approach is used to generate the tetramer **57** is outlined in Scheme 17. The cyclic acetal‐protected β‐O‐4 linking motif model **55** can be synthesized over four steps (28 % overall yield). Dimerization using potassium ferrocyanide similar to that shown in Scheme 12 is used to install the 5‐5 motif in the center of the tetramer **56. 56** can then be deprotected under acidic conditions to give the tetramer **57** (β‐O‐4) (5‐5) (β‐O‐4).

These two related approaches can be very successful as they allow the swift building up of multi‐linking motif model compounds in moderate‐to‐good overall yields. The radical nature of the coupling reaction in the second approach is a drawback as it limits the types of linking motifs that can be formed and also limits the functional group compatibility of the reaction.

**Scheme 18:**
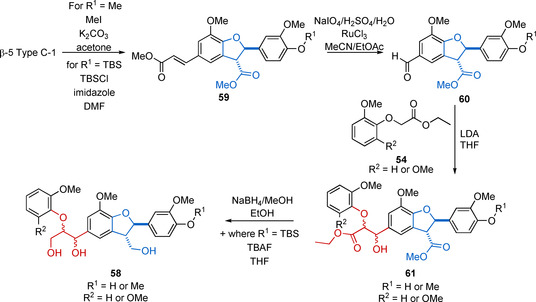
Synthetic route developed by Lahive et al. to the trimeric model compounds (β‐O‐4) (β**‐**5).^56^ TBAF=tetra‐*n*‐butylammonium fluoride.

Others have taken the approach of synthesizing a specific linking motif or a series of linking motifs and then combining them in discrete non‐dimerization reactions to generate the desired oligomeric product. Each linking motif is combined with a functional group or masked functional group, which can be used in subsequent steps to build‐up the desired oligomer. This approach is somewhat more versatile as it does not necessarily result in symmetrical model compounds. This approach has been used to synthesize trimers^58^, ^150^, ^151^, ^152^, ^153^, ^154^ (2 linking motifs (β‐O‐4) (β**‐**1), (β**‐**5) (β‐O‐4) and multiple (β‐O‐4) (β‐O‐4)), and tetramers^151^ (3 linking motifs (β‐O‐4) (β‐O‐4) (β‐O‐4)). An example of this approach reported by Lahive et al.^56^ is shown in Scheme 18 in which a (β‐O‐4) (β**‐**5) model compound of general structure **58** is synthesized. The approach essentially follows the β‐5 Method 1 a approach of dimerizing **21** to generate the β‐5 Type C‐1 as outlined in Scheme 5. This was followed by methylation or *tert*‐butyldimethylsilyl ether (TBS) phenol protection and an oxidative cleavage step to install an aldehyde group. This allowed the application of the Nakasubo method of β‐O‐4 synthesis as outlined in Scheme 1 b, followed by reduction and, if necessary, TBS removal to give access to the desired (β‐O‐4) (β‐5) model compound.

### 3.2 Enzymatic coupling

**Scheme 19:**
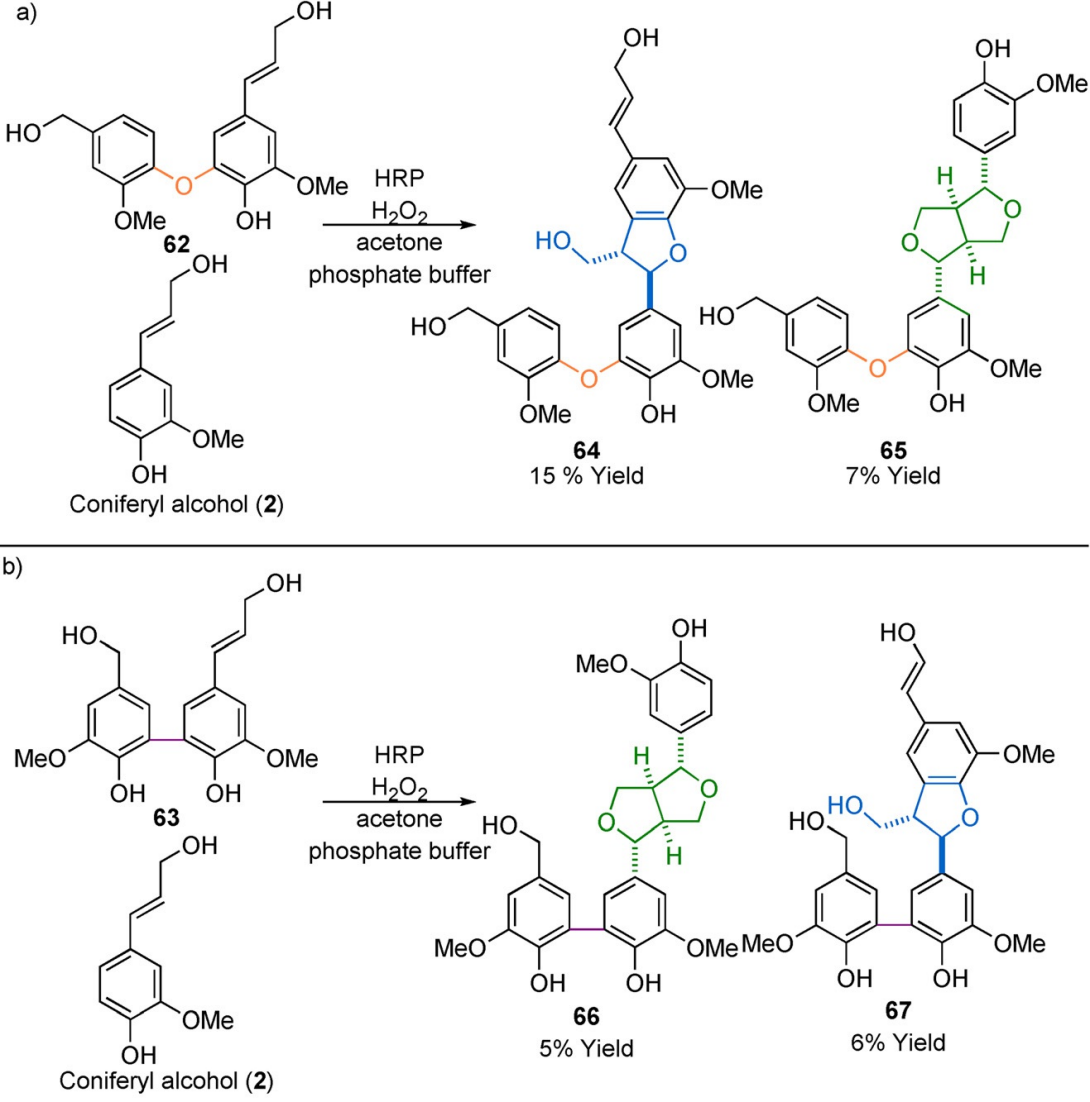
Selection of the products generated via HRP radical dimerization of a) a preformed 4‐O‐5 linked dimer (**62**) with coniferyl alcohol (**2**)^22^, ^23^ and b) a preformed 5‐5 linked dimer (**63**) with **2** 22 Note: not all products identified from the complex mixture formed in these reactions are shown, for example, both reactions produced large quantities of **2** homodimerization.^22^, ^23^

The other general method of multi‐linking motif model compound synthesis is to use HRP to synthesis a large number of products (including dimers and oligomers) in a single reaction.^22^, ^23^ The advantages of this approach are that it can generate a large number of model compounds in a single reaction, which often have highly realistic features when compared with native lignin. The disadvantages are that the purification of each individual compound from the generated mixture is quite challenging, and the compounds are generally isolated in poor yield. Despite these drawbacks, the methodology has been used to remarkable effect in generating 4‐O‐5‐ and 5‐5‐linked model oligomers, primarily trimers, comprised of 5‐5 or 4‐O‐5 model compounds linked with β**‐**5, β‐β, and β‐O‐4 linking motifs (Scheme 19, only β**‐**5 and β‐β are shown). These model compounds were generated from the appropriate 4‐O‐5 (**62**) or 5‐5 (**63**) containing starting compounds that also contained a 4‐hydroxycinnamyl alcohol motif. These starting compounds can then undergo dehydrogenative coupling with (excess) **2** to generate a range of new linking motifs. Scheme 19 shows only a selection of the most interesting products from these reactions. Also formed were β‐O‐4‐, β**‐**5‐, and β‐β‐linked homodimerization products of **2**. All of these products contribute to the complexity of the product mixture, complicating the isolation of products and contributing to the relatively poor yields. Nevertheless, compounds generated via this methodology proved invaluable in the detailed study of multi‐linking motifs in native lignin, using 2D HSQC NMR techniques and detailed studies of the lignin biosynthesis pathways.^22^, ^23^

## 4 Polymeric Models of Lignin

### 4.1 Biomimetic synthetic lignins

The synthesis of model lignin polymers takes the final step in the hierarchy of complexity in relation to the complex polymeric substance that is lignin. Work has been carried out to synthesize model lignin polymers using biomimetic approaches that attempt to replicate the stepwise combinatorial radical coupling of monolignols that occurs during lignification, so called dehydrogenative polymerization (DHP). Depending on the exact conditions used these model polymers can be highly realistic, with a similar complexity to lignin *in planta* or isolated native lignin, including replicating the two‐ and three‐dimensional structure, an attribute that cannot be achieved by individual linking motif model compounds and is unlikely to be fully achieved by oligomeric models. Nevertheless, their complexity reintroduces some of the characterization challenges present for plant‐derived lignins. Additionally, the nature of combinatorial coupling means it is impossible to accurately control the linking motif distribution.

The most common approach to prepare DHP lignins (or DHPs) involves using HRP and hydrogen peroxide to polymerize mixtures of monolignols in buffered solutions as a mimic for the biosynthesis of lignin in nature.^155^, ^156^, ^157^, ^158^, ^159^, ^160^ The advantages of this approach are that it generates the desired complex polymer in one step, and it should, in theory, integrate all the known lignin linking motifs. Two main methods for the polymerization exists the so‐called “zulauf” and the “zutropf” methods. The zulauf method involves the bulk polymerization of monolignols and leads to an overabundance of dimerization products compared to natural lignin. The zutropf method, on the other hand, involves the slow addition of monolignol and hydrogen peroxide solutions to HRP, favoring an end‐wise polymerization process, reducing the proportion of dimerization products. This results in higher molecular weight DHPs compared to the zulauf method.^161^ DHPs produced using either of these methods, however, have lower molecular weights than *in planta* lignin and so an extension of the zutropf method has been developed were a cellulosic dialysis tube containing the HRP is placed in a flask containing the hydrogen peroxide and monolignol solution. The use of dialysis tubing isolates the HRP and growing polymer molecules from the bulk of the mono‐ and oligolignols, resulting in a relatively high concentration of polymer radicals, which thus favors polymer–monolignol over monolignol–monolignol coupling reactions. This method allows for the production of DHPs with molecular weights more akin to that of native lignin.^162^ DHP lignins have found extensive use in studying biological depolymerization processes,^163^, ^164^ particularly due to the ability to ^14^C label them;^165^, ^166^, ^167^, ^168^ in verifying the ability of non‐canonical monolignols to participate on lignification;^169^, ^170^, ^171^, ^172^ and in studying selective depolymerization processes.^173^, ^174^

### 4.2 Non‐biomimetic synthetic lignins

**Scheme 20:**
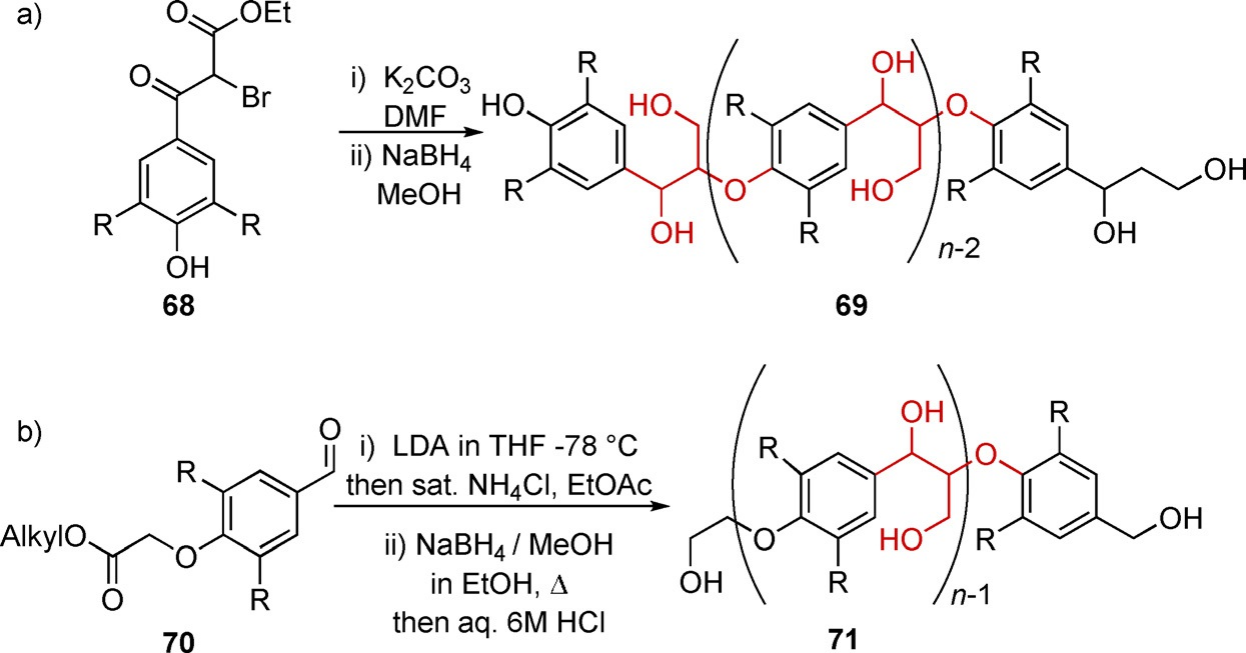
Two examples of synthetic approaches to synthetic lignins containing exclusively β‐O‐4 linking motifs.^176^, ^179^ (R=H or OMe).

Non‐biomimetic approaches have also been thoroughly investigated, resulting in numerous literature methodologies for the synthesis of many kinds of these model polymers. Early lignin model polymers often lacked some aspects of individual linking motif structures^175^ while others consist entirely of a single linking motif, usually β‐O‐4.^176^, ^177^, ^178^ Two general approaches for complete β‐O‐4‐based lignin model compounds are outlined in Scheme 20. In (a) a brominated polymer precursor is synthesized (**68**), which can polymerize under mildly basic conditions. A final reduction step allows access to an exclusively β‐O‐4‐containing model lignin polymer (**69**).^176^ In (b) a bifunctional polymer precursor is prepared with one end containing an ester and the other end an aldehyde (**70**). This compound can then be polymerized by treatment with lithium diisopropylamide, a variation of the Nakasubo method of β‐O‐4 synthesis outlined in Scheme 1 b. Final reduction of this polymer yields an exclusively β‐O‐4‐containing model lignin polymer (**71**).^58^, ^177^, ^178^, ^179^

**Scheme 21:**
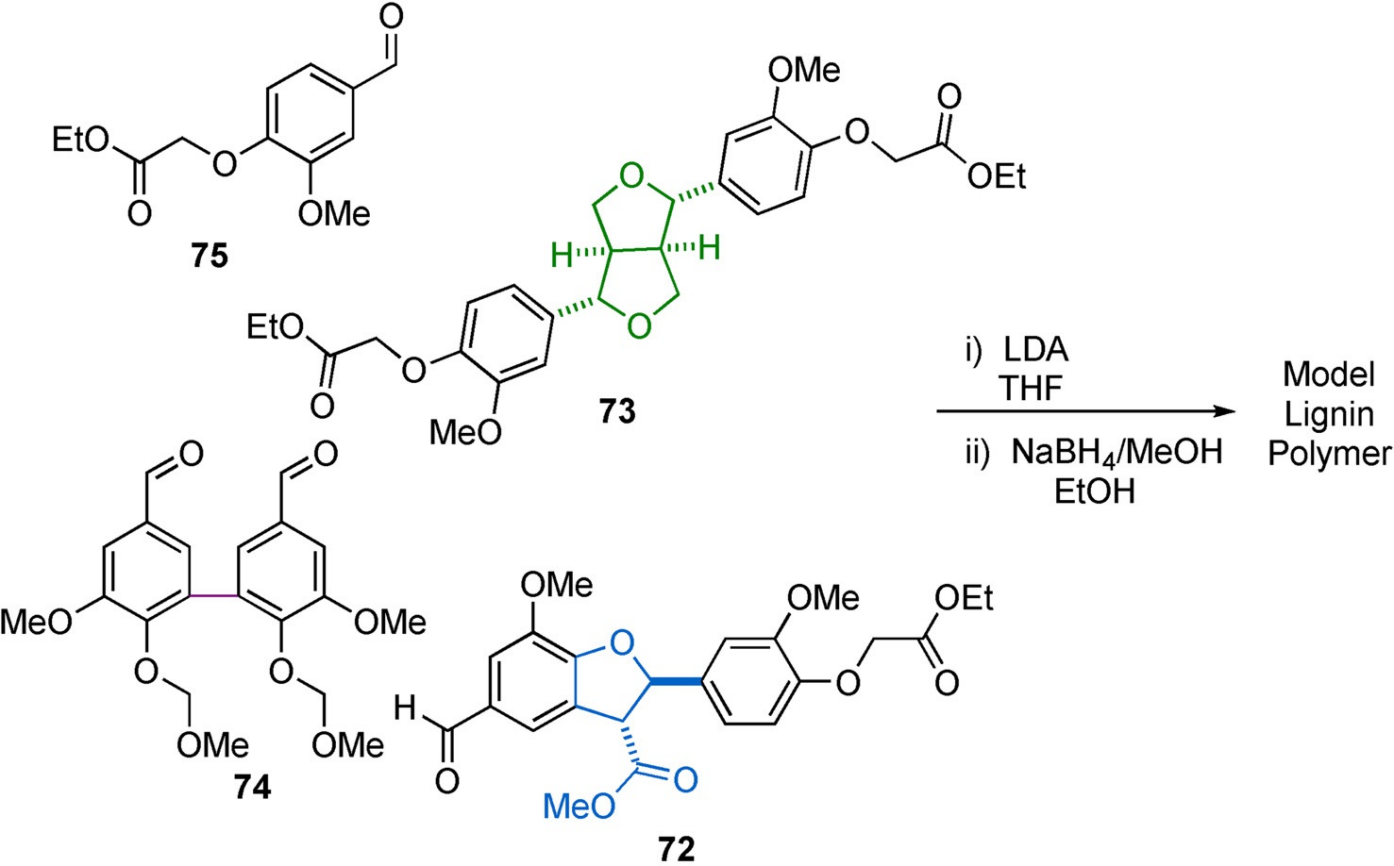
Synthesis of lignin model polymer containing β‐O‐4, β‐5, β‐β, and 5‐5 linked units distributed within its structure.^104^

In more recent years, this methodology has been further developed by Lancefield and Westwood^104^ (Scheme 21) for the synthesis of model lignin polymers that contain β‐O‐4, β‐β, β‐5, and 5‐5 motifs. This was achieved via the synthesis of linking motif models with functional groups, which allow them to participate in an adapted Nakasubo method of β‐O‐4 synthesis. Following a reduction step, a model lignin polymer with complete compositional control can be accessed, making them highly realistic models for lignin.

## 5 Concluding Considerations for Using Lignin Model Compounds for Reactivity Studies

Many of the above models have a great value in aiding lignin structural elucidation by, for example aiding in identifying signals in 2D‐HSQC NMR analysis or by comparison of depolymerization mixture with those from model systems. This has allowed for the identification of the structural motifs within lignin and also of the bonds between lignin and other biomacromolecules, and new structures are still being identified and confirmed to this day.^22^, ^23^, ^180^, ^181^, ^182^, ^183^, ^184^, ^185^ Here, the complexity of the lignin biopolymer needs to be sufficiently matched by the complexity of the model compounds to ensure a good signal overlap is observed in 2D‐HSQC‐NMR spectra. Therefore, complexity is often desired despite the synthetic effort.^22^, ^23^ 2D‐HSQC NMR spectroscopy is indeed the preferred method to analyze lignin as well as larger synthetic model compounds as it circumvents problems with signal overlap in conventional ^1^H and ^13^C NMR spectra and thus gives very detailed structural information. Also, when selective chemical modifications are performed, 2D‐HSQC NMR spectroscopy is the preferred method of analysis to identify structural changes.^72^, ^114^, ^186^, ^187^, ^188^ Problems with the quantification of 2D‐HSQC NMR spectra can also now be circumvented by the development of specific pulse sequences.^189^, ^190^, ^191^ For more specific information on the size of extracted lignins as well as larger model compounds researchers turn to size‐exclusion chromatography, although the molecular weight data obtained highly dependd on the system and standards used in combination with the mobile phase.^104^, ^192^, ^193^ Other techniques can be applied such as MALDI,^194^ but also DOSY NMR.^179^, ^195^ Identification and quantification of specific functional groups in lignin such as ketones, alcohols/phenols, and carboxylic acids such as titrations can be performed using titrations,^196^ FTIR spectroscopy,^20^ or specific reagents in combination with ^31^P and ^19^F NMR spectroscopy^197^, ^198^

The application of smaller (typically dimeric) model compounds differs significantly from those of larger oligomeric and polymeric model compounds. Already simple dimeric model compounds can be applied to excellent effect in the development of new catalytic methodologies for the breakdown of specific linking motifs, identify potential depolymerization products, or to elucidate the effect of chemical treatment on the lignin structure. This is due to the simplified structural characterization and quantification of reaction products. Due to the simpler product mixtures 1D NMR^24^, ^72^, ^80^, ^114^, ^187^, ^199^ and mass spectroscopy methods such as LCMS or GCMS are available that give much greater detail on the chemical structures.^56^, ^188^ Once reaction products have been identified, GC‐FID and HPLC but also ^1^H NMR spectroscopy can be used to monitor reactions over time to give detailed reaction profiles and pathways.^50^, ^56^, ^73^, ^187^

Based on our experience in the field we provide some basic guidelines on what to consider when selecting model compounds for lignin reactivity studies. This should then allow one to select the appropriate model compound and how to access it using the overview provided in this Review. The primary considerations are related to 1) the feedstock used and 2) the stage or depth of the study in relation to the synthetic effort required to access specific highly complex lignin model compounds. These two considerations will be discussed below. This section constitutes studies specifically involving lignin linking motifs for which model compounds are described in this manuscript.

### 5.1 Lignocellulose/lignin feedstock

**Figure 9:**
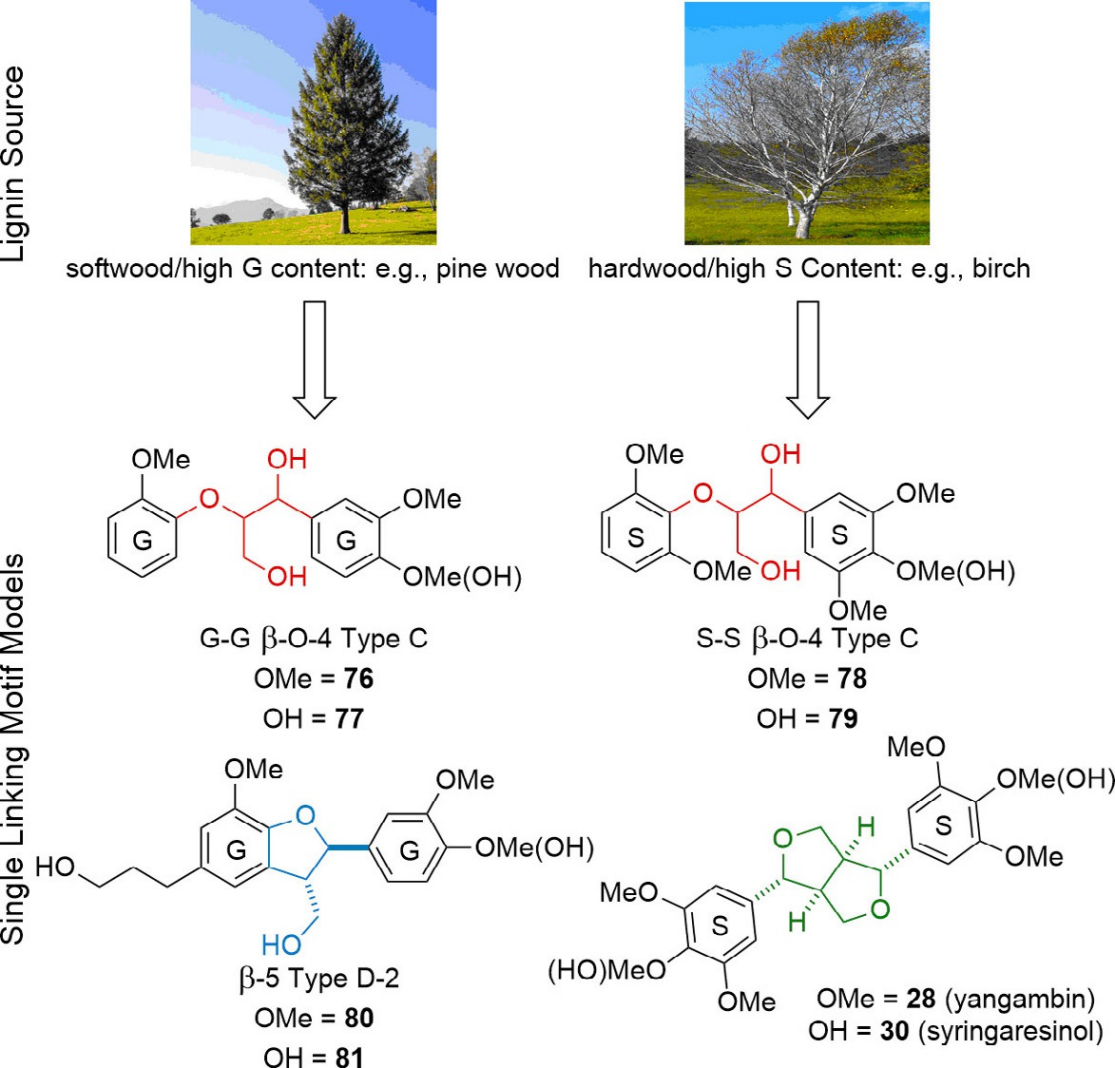
Linking lignin feedstock to model compound choice based on characteristic linking motifs found in relatively high abundance in the source material.

The target lignin feedstock of a study can significantly influence the choice of model compound for reactivity studies. The plant origin of the lignocellulosic biomass can guide model compound selection to represent the lignin structure. The origin determines the ratios of the various aromatic units (S/G/H) as well as the types of linking motifs present in the native lignin. These can differ significantly not only between botanical species but also between the segments of the plant chosen as well as the growth environment and stage of development. Therefore, determining these beforehand can be beneficial while more generic information such as provided in Table 1 of this manuscript can be helpful. This can direct specific substitution patterns of the aromatic rings of the selected model compounds. For example, in a study targeting softwoods that contain almost exclusively G‐type aromatics the corresponding G‐based lignin model compounds can be selected whereas for lignin originating from hardwood and specific grasses S‐type aromatic substitution patterns might be a better match (Figure 9). Examples are β‐O‐4 type C model compounds **76/77** and **78/79** representing such motifs. Although the β‐O‐4 motif is the most abundant in nearly all native plant material, its relative abundance varies and in some species such as grasses γ‐esterified β‐O‐4 motifs can be relatively abundant. The distribution of the linking motifs is often also related to aromatic substitution pattern due to the nature of lignin biosynthesis.^9^ Another factor to bear in mind is that the distribution of β‐O‐4 diastereomers in lignin is under chemical control. In general, this means for softwood G‐type lignins the *syn*/*anti* ratio is ≈1:1 and in hardwood S‐type lignins the ratio is closer to ≈1:3, with S units favoring the formation of *anti* isomers.^16^, ^43^ Also, typically, G‐type lignins are relatively more abundant in β‐5 linking motifs whereas S‐type lignins are relatively more abundant in β‐β linking motifs.^200^ If a study is focusing on the effect of a chemical conversion methodology on the lignin structure, this can thus guide model‐compound selection to structures **80/81** or **28/30**. There are specific analytical methods to determine the S/G/H ratio and relative abundance of linking motifs. For example, whole‐cell NMR methods can be used to determine both but require milling and solvation of the source material and access to cryo‐probe‐equipped NMR instruments and/or significantly extended NMR measurement times.^201^, ^202^ Alternatively FT‐Raman,^203^ FTIR spectroscopy^204^ and pyr‐GC^205^, ^206^, ^207^ can be used to determine S/G/H ratios with proper calibration.

The considerations above are important when dealing with lignin as part of the biomass material, for example, when converting lignin as part of the lignocellulose material by fractionation methodologies of the lignin‐first type.^208^, ^209^, ^210^, ^211^ However, in many cases research is done on specific fractionated lignin streams. Here, the fractionation methodology can significantly alter the structure of the lignin extracted from the original biomass source. For example, specific fractions of lignin are often extracted, which results in different H/G/S ratios compared to the native lignin material. This effect is often minor compared to the effect of the processing on the native linking motifs in lignin. Indeed, the β‐O‐4 linking motif is the most common motif found in natural lignin and is, therefore, the main focus of study. However, of the linking motifs discussed in this Review, it is, apart from the α‐O‐4, also the most labile. Thus, one should consider that the β‐O‐4 motif is also affected by most lignin extraction strategies. In technical lignins obtained from current large‐scale lignocellulosic biorefineries such as the Kraft process used in the paper industry, the amount of native β‐O‐4 linking motifs is typically low due to the harsh conditions leading to its breakdown and/or modification. Other linking motifs, primarily C−C‐containing motifs obtained via recondensation processes, become more dominant.^3^, ^52^, ^212^ Therefore, one should appreciate that selecting the β‐O‐4 linking motif as being representative of the primary linking motif in all lignins is not correct. If one wishes to study effective catalytic methodology to depolymerize technical lignins using β‐O‐4 model compounds is not going to be helpful.^213^, ^214^ In such cases C−C‐linked aromatic dimers with alkyl or 5‐5 linking motifs might be more suitable.^215^ Such models are not the focus of this Review as the actual identity of the linking motifs in such technical lignins is highly diverse and for a large part unknown.^52^ More analysis of technical lignins is required to guide the synthesis of a new generation of model compounds for use in the valorization of these recalcitrant lignins. For structural determination of these newly formed linking motifs under pulping process conditions, reactivity studies using model compounds representing the native lignin structure, as discussed in this Review; are highly beneficial.^24^, ^52^ Overall, this means that analysis of the lignin material by for example NMR or IR spectroscopy can be extremely useful to provide insight into the structure of lignin and how relevant the use of model compounds representing the native linking motif content can be.^20^, ^52^

### 5.2 Technology development levels

**Figure 10:**
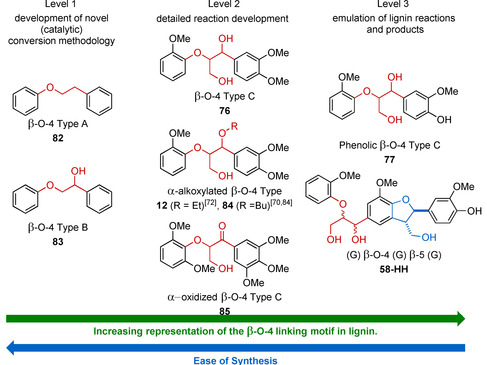
Selection of lignin model compounds ordered in relation to their complexity and degree of representativeness of real lignin.

A second consideration for the choice of model compound is at what stage of development the study currently is and what insight is being targeted. In relation to studies that involve chemical reactions on lignin, be they novel depolymerization pathways or specific modification methodologies, or studying structural effects from a biomass processing and fractionation perspective, there is a balance to be struck between the desired synthetic effort and the depth of the insight that can be gained. Here, we would like to distinguish three levels of development that link to the appropriate type of model compound selection (Figure 10). These levels are the result of our experience in studying lignin reactivity in relation to the catalytic breakdown of its structure as well as selective chemical modification. Increasing levels represent an increase in the synthetic effort required to access the appropriate model compounds while each level results in different levels of information regarding lignin reactivity and how this can be used to develop lignin conversion methodologies.

The first level is the development of novel (catalytic) conversion methodology. In this case, screening of different conditions and catalyst is likely the focus. Thus, high‐throughput is often desired or at least a swift answer to whether lignin‐like C−O or C−C bonds can be broken. Relatively simple model compounds are likely more suitable in this case owing to the minimal synthetic effort required to access them. Additionally, relatively more straightforward analysis is possible, and reactions are likely to proceed without the intrusion of many possible complicating side reactions. This can be especially useful when substrates and products can be readily analyzed by GC (especially GC–MS), which gives easy access to quantitative data to compare and assess development directions. An example of a relatively easy to access model compound is the β‐O‐4 Type A model **82**. In such studies, the choice for a relevant aromatic substitution pattern is typically also considered less critical as the focus is primarily on activation of the β‐O‐4 C−O bond.^216^, ^217^, ^218^, ^219^, ^220^, ^221^ Alternatively, a β‐O‐4 Type B model such as **83** can be used if more information on the reaction product is desired^42^, ^222^, ^223^, ^224^, ^225^, ^226^, ^227^, ^228^, ^229^, ^230^ or if the breaking of the β‐O‐4 C−O bond is envisioned to be facilitated by the oxidation of the benzylic alcohol in the α‐position.^17^, ^231^, ^232^, ^233^, ^234^, ^235^, ^236^ For the breaking of C−O bonds in general, sometimes even more simple commercially available model compounds such as benzyl phenyl ether are used for initial catalyst development.^237^, ^238^

The second level involves more detailed reaction development. Here, the actual feasibility of the intended reaction on lignin, targeting a specific lignin chemical motif, can be tested. This can be accompanied by detailed mechanistic insight and identification of major reaction products as well as potential side products that might arise from the reaction when applied to lignin. At this point in methodology development, it would be advisable to have the appropriate lignin‐linking motif fully represented, including all functional groups and preferably also to have the correct substitution patterns on the aromatic rings. Returning to the example regarding insight into the reactivity of the β‐O‐4 linking motif, for this stage, an appropriate model compound may be a β‐O‐4 Type C model such as **76**. Here, all β‐O‐4 linking motif functionalities found in native lignin are present. This allows for the monitoring of the fate of all fragments released upon the application of a developing methodology that leads to the breakdown of this motif. This will lead to aromatic products with similar chemical structures to those expected from the breakdown of lignin. This typically comes from cleavage of the β‐O‐4 C−O aryl bond releasing a phenolic fragment,^222^, ^239^, ^240^ but β‐O‐4 Type C can also offer insight into Cα−Cβ bond cleavage that would not be observed in β‐O‐4 Type A and Type B model compounds.^82^, ^241^, ^242^, ^243^ Additionally, modification of the lignin model compounds to represent the chemical modification of lignin from a specific source can be taken into account at this stage. For example, β‐O‐4 Type C model compounds that have a ketone at the α‐position such as **85** are readily accessible and represent the β‐O‐4 linking motif in lignin that has been selectively oxidized at the benzylic position.^58^, ^69^, ^80^ Additionally, modifications arise from fractionation, such as organosolv extraction with alcohols, which incorporates alkoxy groups at the α‐position. Models for these modified structures, such as compounds **12** and **84**, can be accessed.^70^, ^72^, ^84^ It is during this stage of reaction development that the effects of model compound stereochemistry may become apparent upon detailed analysis of reactions. Working with, for example, diastereomeric mixtures of β‐O‐4 Type C models it is, in principle, possible to observe reactivity differences between diastereomers if analysis of changes in the diastereomeric ratios during the course of reactions is carried out. Such findings may prompt more detailed mechanistic studies, in which case model compounds synthesized via the routes discussed previously giving diastereomerically or enantiomerically pure model compounds can be employed. Studies at this level thus already give very good insight into the reactivity of the specific linking motif and what type of products are expected.

The third level seeks to emulate lignin reactions and products but also avoid the complete heterogeneity of lignin itself. Such a study can focus on the phenolic nature of products obtained through C−O bond cleavage methodologies, using β‐O‐4 Type C model compounds such as **77**. Larger model structures of lignin, such as **58‐HH**, which combine multiple linking motifs in sequence and be used to directly predict and identify products from reactions with real lignin. The focus here is twofold, firstly to determine the fate of linkage motifs other than those upon which the methodology was developed. Indications of the reactivity of previously unstudied linkage motifs can be obtained by using some of the simpler model systems discussed previously; however, by using a model such as **58‐HH** the second objective can also be achieved, which is getting reaction products that exactly match those present in a lignin depolymerization mixture such a possible dimeric structures obtained from the reactivity differences of various linking motifs. Here, for example, it can be shown how other linking motifs might affect the reactivity of neighboring linking motifs as well as how these linking motifs influence the applied catalyst. Additionally, oligomeric model compounds can be used for reproducing challenges of working with lignin itself regarding solubility, product recovery, and analysis.^22^, ^23^, ^50^, ^56^, ^58^, ^243^, ^244^

**Figure 11:**
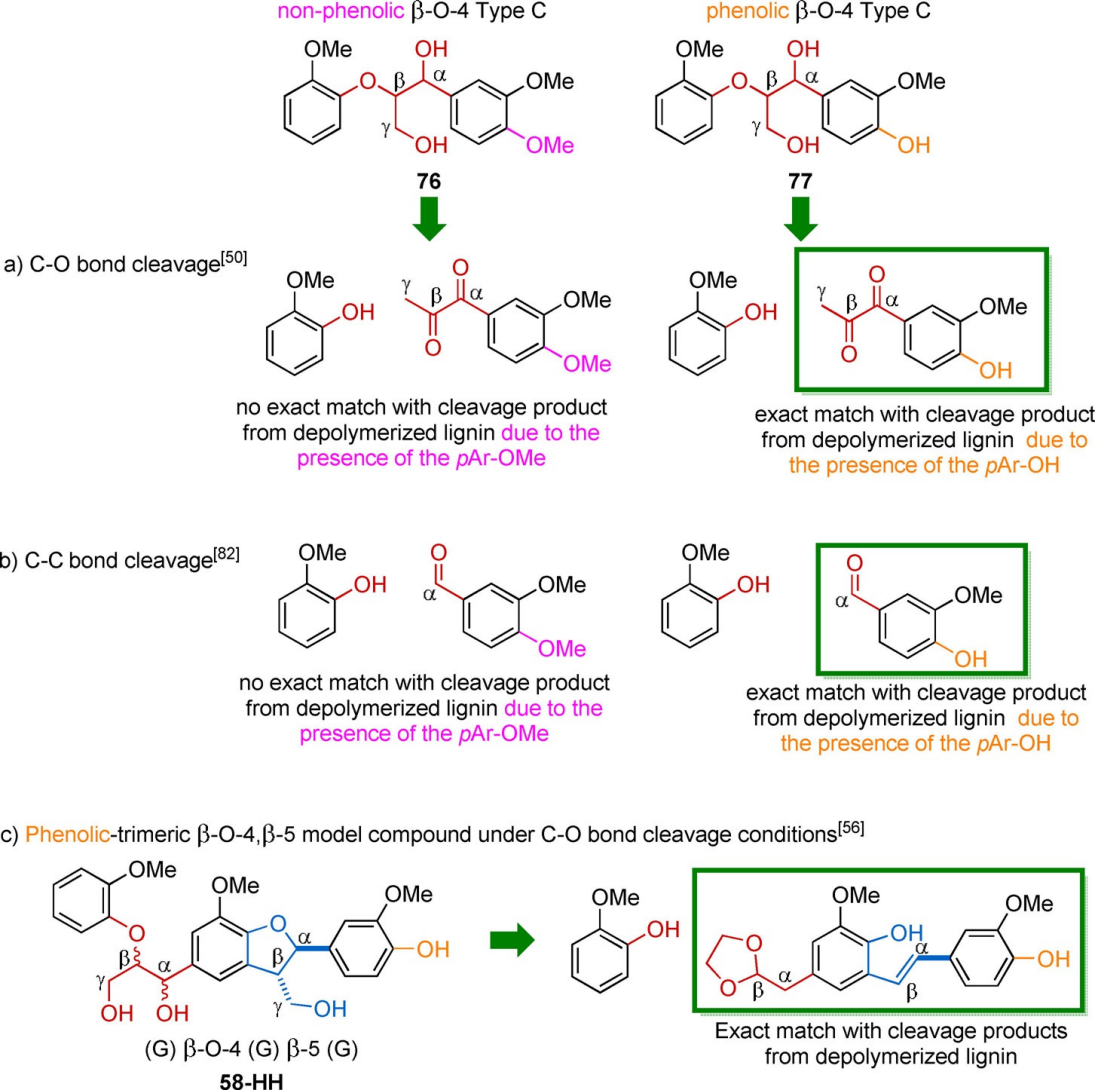
Selection of model compounds and their relation to actual lignin depolymerization reaction products, contrasting phenolic and non‐phenolic model compounds using methodology developed by a) Stahl and co‐workers,^50^ b) Bolm and co‐workers,^56^ and c) Barta and co‐workers.^82^

In the case of research into the catalytic breakdown of lignin, there are limitations to the use of dimeric model compounds such as β‐O‐4 Type C model compound **76** (Figure 11). As lignin is a polymer, compound **76** cannot give the exact reaction products that would be observed from the degradation of the lignin structure as the 4‐O‐position is methylated, therefore, typically, a phenolic compound such as β‐O‐4 Type C model compound **77** (Figure 11) would be required. If, for example, in the sequence of a lignin chain a β‐O‐4 linking motif is flanked by two more β‐O‐4 linking motifs and a C−O bond‐cleavage methodology is applied to this sequence, ultimately, exclusively phenolic products will result. In this sense, C−O bond cleavage of the β‐O‐4 linking motif of compound **77** results in the formation of compounds that can be an exact match with those that come from lignin itself, which is not the case for the non‐phenolic compound **76** (Figure 11 A). It is important to note that, as lignin depolymerization is in most cases, unlikely to be an exclusively step‐wise process involving phenolic end groups, compound **76** is still a valuable model for studying the reactivity of internal (non‐phenolic) lignin units during depolymerization processes. An additional advantage of using phenolic models such as **77** is that the stability of the lignin degradation products can be assessed under the conditions at which they will be formed during lignin depolymerization to ensure repolymerization is not occurring. If a C−C bond‐cleavage strategy is being utilized, phenolic models are also useful in determining the exact products that will be derived from lignin, but this is only the case if the β and γ carbon atoms following the cleavage of the linking motif are themselves removed to reveal a phenol, as is seen in Figure 11 B.^82^ If this is not the case, tetrameric β‐O‐4 linking motif model compounds would be required^151^ or authentic standards of suspected products need to be synthesized for authentication and calibration purposes.^243^ For the identification and quantification of lignin depolymerization products derived from other linking motifs, the β‐5 for example, trimeric β‐O‐4,β‐5 model compounds such as **58‐HH** (Figure 11 C) can be used.^56^ This is especially useful when dealing with minor products in highly complex depolymerization mixtures. The use of larger oligomeric model systems are also of use in this respect and can also provide great insight and confirmation regarding the structures within lignin and also structural modifications in lignin.^22^, ^23^, ^58^, ^114^

Studies at all of these levels provide different insight. However, combining information from all levels offers the most powerful approach. Initial reactivity testing with new precious catalytic material can likely better be performed at the first level at which the analysis of starting material and products are not the limiting factor. On the other hand, further development towards understanding actual reactivity on lignin requires research at the second level preferably combining results from model compounds representing different lignin linking motifs present in the source material. Finally, identification of reaction products can be greatly facilitated by research at the third level, aiding product identification and simulation of the behavior of the oligomeric/polymeric material. This is not necessarily the order at which such studies should take place. Feedback from each study as well as the studies on lignin itself might pose research questions that can be answered by studies at each of these three levels. Finding a right balance between model compound reactions at the right level and relating model compound structures appropriately to the source material of interest should as a whole yield insight that can bring lignin valorization technology to the next level.

## Conflict of interest

*The authors declare no conflict of interest*.

## Biographical Information

Ciaran W. Lahive obtained a degree in Chemistry of Pharmaceutical Compounds from University College Cork, Ireland, and went on to complete his PhD in 2018 as a Marie Curie Research Fellow within the innovative training network (ITN) “SuBiCat” at the University of St Andrews, United Kingdom. Under the supervision of Paul Kamer, his doctoral research focused on lignin model compound development and the catalytic depolymerization of lignin. During his studies he carried out a secondment in the group of Katalin Barta at the University of Groningen. He is currently a postdoctoral researcher in the group of Erik Heeres, working within the Engineering and Technology Institute Groningen (ENTEG). His present research focuses on the efficient and sustainable production of chemicals derived from biomass.

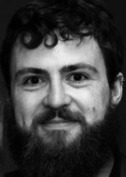

## Biographical Information

Paul Kamer obtained a degree in biochemistry at the University of Amsterdam, Netherlands, and did his PhD in physical organic chemistry at the University of Utrecht, Netherlands. As a postdoctoral fellow of the Dutch Cancer Society (KWF) he carried out postdoctoral research at the California Institute of Technology, USA, and the University of Leiden, Netherlands. He was appointed Lecturer at the University of Amsterdam and full Professor of homogeneous catalysis in 2005. In 2005 he received a Marie Curie Excellence Grant and moved to the University of St Andrews. In 2017 he moved to the Leibniz Institute for Catalysis in Rostock, Germany. His current research interests are (asymmetric) homogeneous catalysis, biocatalysis, combinatorial synthesis, and artificial metalloenzymes.

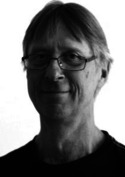

## Biographical Information

Christopher Lancefield received his PhD from the University of St Andrews in 2015. After postdoctoral positions at the University of St Andrews and Utrecht University, he is currently a Leverhulme Early Career Research Fellow in the School of Chemistry at the University of St Andrews. His current research interests are mainly focused on understanding lignocellulose degradation in nature, lignin model compound development, and the catalytic conversion of biomass streams.

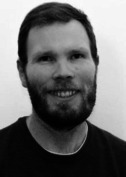

## Biographical Information

Peter J. Deuss completed his studies at the University of Amsterdam and thereafter joined the group of Paul Kamer at the University of St. Andrews as a PhD student. He obtained his degree in 2011 and after working at the medical research council (MRC) UK, Laboratory of Molecular Biology Cambridge he moved to the University of Groningen where, after postdoctoral work in the groups of Katalin Barta and Erik Heeres he started in 2016 as a tenure‐track assistant professor in green and smart biomass processing at the chemical engineering department of the Engineering and Technology Institute Groningen (ENTEG).